# Effects of the Soothe Vision well-being tool on university students’ mood: a pilot study

**DOI:** 10.1007/s12144-025-07649-7

**Published:** 2025-03-31

**Authors:** Asnea Tariq, Yumeng Yang, Ziqiao Liu, Siu Ching Wong, Elaine Gray, Angela L. McLaughlin, Caden J. Arthur, Stella W. Y. Chan

**Affiliations:** 1 Charlie Waller Institute, School of Psychology & Clinical Language Sciences, University of Reading, Earley Gate, Whiteknights, Reading, RG6 6ES UK; 2 School of Health in Social Science, University of Edinburgh, Edinburgh, UK; 3 Port Glasgow High School, Port Glasgow, UK

**Keywords:** Soothing images/videos, Well-being, Self-help intervention, University students, Depression, Anxiety

## Abstract

The *“Soothe Vision”* well-being tool was designed through co-production with young people, combining soothing images from *Project Soothe* and literary quotes and music to produce a set of soothing videos. The present research was a pilot study evaluating its effects on mood states in university students. Specifically, it examined if the *Soothe Vision* tool (intervention group) was more effective than viewing the soothing images from *Project Soothe* alone (control group) in producing positive mood changes. This study was conducted online with 151 Chinese university students (age *M* = 22.77; *SD* = 0.23) both in and outside China. Standardised measures were used to assess the symptoms of depression, anxiety, personality traits and loneliness at the baseline phase. Changes in mood states were measured before and after the intervention. Repeated measure ANOVAs indicated that both groups showed reduced negative mood states (i.e., negative affect, depressive and anxious mood) and an increased serenity affect; however, there were no group differences. Correlation analyses suggested that higher levels of depressive and anxiety symptoms at baseline were associated with a greater reduction in depressive and anxious mood. Participants with a higher neuroticism score reported larger reductions in anxious mood states. These preliminary findings suggest that imagery-based tools/interventions can be beneficial in increasing positive mood and reducing negative mood in students, particularly in those with higher levels of baseline depressive and anxiety symptoms as well as those with higher vulnerability by virtue of neuroticism.

**Supplementary Information:**

The online version contains supplementary material available at 10.1007/s12144-025-07649-7.

## Introduction

### Rising mental health challenges among university students and emergence of Soothe Vision as an innovative intervention

The prevalence of mental health conditions among higher education students has risen significantly in recent years (Lewis & Bolton, 2023; Campbell et al., 2022), with the number of students reporting mental health crises has increased sevenfold (Lewis & Bolton, 2023). The Insight Network Survey (2020), which assessed 37,500 students across 147 universities in the UK, revealed that nearly one in five students currently have a mental health diagnosis. Alarmingly, nearly half of the students reported experiencing serious psychological issues requiring mental health support, representing an increase from 1 in 3 students in the same survey conducted two years before (Pereira et al., 2019). Similarly, a recent survey by the mental health organisation *'Student Minds'* found that 57% of students self-reported experiencing mental health difficulties, with 27% being clinically diagnosed with a mental health condition (Lewis & Bolton, 2023).

Amid these alarming trends, the need for innovative, accessible, and scalable mental health interventions has become increasingly urgent (Ghosh et al., 2023). This study therefore piloted and evaluated *“Soothe Vision”,* a well-being tool initially developed through co-production and participatory research with young citizen scientists. This tool was designed to enhance emotional resilience through a combination of soothing images, music and inspirational literary quotes. This intervention built on evidence from *Project Soothe*, which has successfully demonstrated the potential of soothing images to reduce psychological distress (MacLennan et al., 2023; Witten et al., 2022; Ryynanen et al., 2023). By combining multiple sensory modalities, *Soothe Vision* aimed to provide a more immersive and effective soothing experience (Shuman et al., 2016). Furthermore, the use of digital technologies in this tool aligns with contemporary research identifying digital platforms as accessible strategies tailored to meet the diverse mental health needs of university students (Ruini et al., 2024).

University students constitute a substantial proportion of the vulnerable age group affected by mental health challenges (Thompson et al., 2021), with depression and anxiety recognised as the most prevalent and disabling condition in this population (Yu & Hu, 2022; WHO, 2021). Recent meta-analyses reported a prevalence rate of 25% for depression (Sheldon et al., 2021) and 39.65% for anxiety disorders (Ahmed et al., 2023). Furthermore, psychological distress among university students is associated with negative education, social, and economic outcomes, including an increased risk for suicide attempts, drug abuse, self-harm, academic underperformance, and higher incidences of university drop-outs (Lewis & Bolton, 2023; Yu & Hu, 2022; Campbell et al., 2022). The transition to university often involves significant life changes, such as moving away from home, financial burdens, and academic pressures, exacerbating these challenges (Association of Colleges, 2023).

### Loneliness and personality traits as determinants of university students’ mental health

Among the various contributing factors to poor mental health in this population, *loneliness* is particularly noteworthy. Loneliness is significantly associated with mental health conditions, including anxiety, depression and suicidal ideations (Hards et al., 2021). Research has shown that loneliness is a common consequence of the transition to university (Jaud et al., 2023) and is linked to poorer mental health outcomes (Campbell et al., 2022; Richardson et al., 2017). Additionally, the multifaceted nature of loneliness, influenced by social, psychological, and environmental factors, highlights the complexity of addressing this issue (Korzhina et al., 2022; Yanguas et al., 2018). An integrative review identified four primary contributors to loneliness in adolescents and young adults, including physical and mental health challenges, adverse personal factors such as shyness, ineffective coping, and bullying, as well as transitional phases like entering university (Korzhina et al., 2022). Additionally, the rise of digital technology and excessive social media usage has been associated with heightened feelings of loneliness, emphasising the need for nuance interventions (Nakshine et al., 2022).

In addition to loneliness, personality traits such as *Neuroticism* and *Extraversion* have been recognised as vulnerability factors influencing mental health among university students (Yu & Hu, 2022; Lewis & Cardwell, 2020). Neuroticism, characterised by a tendency towards negative emotionality, is strongly associated with higher levels of anxiety, anger, irritability, and fear (Shokrkon & Nicoladis, 2021). Individuals with high neurotic traits often demonstrate poorer stress responses, increased emotional reactivity, and an amplified sense of insecurity, making them particularly susceptible to mental health challenges (Shokrkon & Nicoladis, 2021). Conversely, extraversion, associated with traits such as sociability and assertiveness, has been linked to greater resilience, enhanced well-being, and positive mental health outcomes (Yu & Hu, 2022). Extraverted individuals are more likely to thrive in social interactions and build supportive networks, which can act as buffers against mental health issues (Yu & Hu, 2022). These findings align with broader research on personality traits within general and university populations, with high neuroticism and low extraversion being consistently identified as significant predictors of mental health problems, including depression and anxiety (Yu & Hu, 2022; Oltmanns et al., 2022; Lewis & Cardwell, 2020; Vinograd et al., 2020).

While extraversion is conventionally associated with favourable outcomes, recent studies highlight its potential downsides when elevated to extreme levels. Excessive extraversion may lead to dominant, impulsive, or attention-seeking behaviours, impacting social relationships and contributing to interpersonal conflicts (Deng et al., 2021). These behaviours may also increase vulnerability to emotional exhaustion and burnout, undermining the protective effects typically attributed to extraversion (Zee et al., 2013). Furthermore, extremely extroverted individuals may experience diminishing social returns, leading to unmet expectations and depressive emotions due to heightened energy expenditure and unfilled social needs (Deng et al., 2021). Meta-analytical findings also reveal that higher neuroticism correlates with greater psychological vulnerability, while higher extraversion is associated with improved therapeutic outcomes and resilience (Bucher et al., 2019).

### The impact of mood states on university students’ mental health

Affective states, encompassing emotions, feelings, impulses and mood, are another pivotal factor significantly influencing the mental health of university students (Gross et al., 2019). Mood states, defined as transient emotional conditions, manifest as positive or negative affect and interact with external stressors to shape psychological responses (Searight & Montone, 2017; Zautra et al., 2005).

*Positive affect*, characterised by joy, energy, concentration, and enthusiasm, is associated with resilience, adaptive coping, and improved academic performance, social engagement, and overall quality of life (Chaudhry et al., 2024; Plys & Desrichard, 2020). Conversely, *Negative affect*, marked by distress, anger and fear, contributes to heightened stress reactivity, diminished coping, and greater psychological vulnerability, often leading to academic and social challenges (Plys & Desrichard, 2020; Tian et al., 2023).

Besides, *depressive* and *anxious* mood states are also particularly associated with adverse mental health outcomes. Depressive mood states manifest as persistent sadness, feelings of hopelessness, and diminished interest or enjoyment in activities, which are symptomatic expressions of depression (Boyle et al., 2015). Contrary to this, anxious mood states are often characterised by excessive worry, restlessness, irritability, and tension, accompanied by physical and somatic symptoms (Rossi & Pourtois, 2011). Among university students, these mood states can impair concentration, reduce motivation, and exacerbate feelings of isolation and loneliness. This affects their social relationships and contributes to academic underperformance and, in severe cases, increased dropout rates (Campbell et al., 2022; Mofatteh, 2021).

### Global and cultural contexts of mental health support for university students

Mental health challenges among university students are a global phenomenon; however, the experiences and institutional support systems available to students vary significantly across countries and cultural contexts (Campbell et al., 2022). While many universities provide free access to short-term counselling, psychotherapy and essential health services, a substantial proportion of students with psychological disorders do not utilise these services (Priestley et al., 2022; Eisenberg et al., 2012). Barriers such as stigma, reluctance, and skepticism about treatment efficacy have been identified as potential deterrents to help-seeking behaviours (Papadatou-Pastou et al., 2017; Eisenberg et al., 2012). Global estimates showed that 60–80% of students do not or cannot access professional mental health support (Mei et al., 2020; Eisenberg et al., 2012). The problem seems to be even worse in China, with nearly 92% of university students reportedly having never sought mental health care (Ning et al., 2022; Liu et al., 2016).

The inability of Chinese university students to access mental health services can be attributed to several factors, including pervasive stigma and insufficient mental health literacy (Ning et al., 2022). A qualitative synthesis involving 150 university students across six different institutions identified additional barriers, such as distrust of campus counselling services, misconceptions about mental health, and logistical challenges in accessing professional care (Ning et al., 2022). Cultural attitudes also play a role, with young people in China often lacking awareness of the underlying factors associated with mental health issues (Shi et al., 2020; Wong et al., 2021). Further, mental health services in China are predominantly provided at specialised hospitals, which are primarily over-stretched and face shortages of trained professionals (Wong et al., 2021; Shi et al., 2020). Also, the financial burden makes it harder for students to seek private psychological support, as such services are typically not covered by public welfare or insurance schemes (Shi et al., 2020).

The organisational structure of university mental health services in China further exacerbates the problem. Approximately 78% of these services are managed by the student affairs offices, which often prioritise other aspects of education over mental health education and support (Ning et al., 2022). A synthesis of 17 studies highlights additional barriers, including tendencies towards self-reliance, reliance on informal support systems, diminished perceived need for professional assistance, familial opposition, and concerns about burdening families (Shi et al., 2020). In light of these challenges, it is imperative to explore and implement alternative strategies to address the unmet mental health needs of university students in China.

### Evidence-based therapeutic strategies and innovations in mental health interventions

Evidence-based therapeutic approaches, such as *Cognitive Behaviour Therapy* (CBT) and *Acceptance and Commitment Therapy* (ACT), have been widely employed in the past to overcome mental health problems among University students (Ning et al., 2022). Despite their effectiveness, significant barriers to accessing mental health support– such as stigma, lack of awareness, and logistical challenges– contribute to a persistent gap between treatment needs and availability for young people (Bennett et al., 2019). To bridge this gap and enhance therapeutic impact, digital single-session interventions have emerged as scalable, accessible alternatives, demonstrating efficacy comparable to traditional, long-term therapeutic approaches (Ghosh et al., 2023).

Recent advancement in digital emotion regulation tools have further expanded the scope of mental health interventions, emphasising the therapeutic potential of visual and auditory stimuli in managing mood symptoms like depression and anxiety. For instance, music therapy has been shown to reduce anxiety by activating the brain’s reward systems and modulating stress response (Zaatar et al., 2023; Witten et al., 2022). Similarly, imagery therapy, employing soothing visualisations, has been shown to significantly help reduce physiological stress markers and improve emotional well-being (Holmes et al., 2008). Besides, Virtual Reality (VR) therapy has also emerged as an innovative and promising intervention in emotional regulation, utilising immersive environments to support mental health treatment (Montana et al., 2020). Research suggested that VR therapy significantly reduces negative emotions and promotes relaxation by engaging users in multisensory stimuli (Montana et al., 2020; Maples-Keller et al., 2017).

Building on these advancements, one promising innovation in mental health support is using *imagery* and *imagery-based therapeutic techniques*. These methods have also proven effective as early intervention and preventive strategies, particularly for reducing symptoms of depression and anxiety while promoting well-being among young people (Pile et al., 2021). Soothing imagery, often integrated into compassion-focused therapy (CFT), has been shown to activate the soothing system, fostering emotional regulation and reducing psychological distress (Gilbert, 2009; Neff, 2003). Empirical evidence suggests that imagery-based interventions enhance feelings of calmness and emotional resilience (Bratt et al., 2020; Vidal & Soldevilla, 2023).

### Soothe Vision tool: an extension of Project Soothe

*Project Soothe* (http://www.projectsoothe.com/), inspired by the theories and practice of CFT, is an ongoing research and public engagement project that has collected a global repository of soothing images contributed by individuals aged 12 and above. The project’s primary aim is to develop a bank of soothing images that can be utilised in evidence-based psychological research to help people self-soothe during periods of psychological distress (MacLennan et al., 2023). Our empirical evidence has consistently demonstrated that these soothing images are effective in reducing negative affect and depressive and anxious mood states while enhancing the serenity effect across different age groups, including both adults (Witten et al., 2022) and adolescents (Ryynanen et al., 2023).

Recently, Project Soothe completed a new co-production initiative with Young Citizen Scientist teams, who were recruited to co-design and co-evaluate innovative well-being tools (https://youth.projectsoothe.com/). Among these innovations is the *Soothe Vision* tool, developed by a team of young citizen scientists from a mainstream secondary school under the leadership of the author CJA. Soothe Vision uniquely integrates soothing images with inspirational literary quotes and music to create immersive video experiences. This multisensory design aims to amplify the therapeutic effects of soothing images by incorporating auditory and linguistic elements, thereby providing users with a richer and more impactful soothing experience than viewing images alone.

The development of the Soothe Vision tool was informed by the lived experiences of the young contributors, who drew upon their personal strategies for coping with everyday stress. This design is further supported by existing research highlighting the efficacy of inspirational words in stimulating positive emotional states (Stafford et al., 2010) alongside robust evidence for the mood-enhancing effects of music therapy in individuals aged 12 to 21 (Shuman et al., 2016; Thomson et al., 2014). Combining these elements, Soothe Vision offers a novel, user-centred approach to emotional regulation, highlighting the power of multisensory stimuli to promote positive mood states.

#### Aims of the present study

Taken together, this was a proof-of-concept study aiming to evaluate if this newly developed *Soothe Vision* self-help tool would positively change mood states. As a secondary aim, this study examined if individual differences in baseline symptoms of depression and anxiety, personality traits (neuroticism and extraversion), and loneliness level would influence the mood effects. Specifically, this study sought to test the following hypotheses:

##### Primary hypotheses

1. Both the *Soothe Vision* well-being tool (Intervention group) and viewing soothing images only (control group) will significantly increase positive mood states (i.e., positive affect, serenity affect) and decrease negative mood states (i.e., negative affect, depressive mood states, anxious mood states) from before to after the intervention.
2. The above-hypothesised mood effects will be larger in the intervention group than in the control group.

##### Secondary hypothesis

3. Baseline levels of depression, anxiety, loneliness, and personality traits will influence the extent of mood changes following exposure to soothing images (in both conditions). Due to a lack of previous research, however, it was deemed appropriate to take an exploratory approach and test this hypothesis without specifying the direction of the associations.

## Methods

### Design

The present research adopted a between-subjects repeated measure experimental design comparing the effectiveness of the newly developed well-being tool *Soothe Vision* (intervention condition) and viewing soothing images alone (control condition) in producing mood changes at two-time points (pre- vs. post-intervention/control). The study was approved by the relevant University Research Ethics Committee. The experiment was conducted online through Qualtrics, a secure survey platform approved by the University Research Ethics Committee. All participants, providing informed consent, were randomly assigned to either the intervention or control condition using a programmed function of the platform. Participants’ baseline levels of depression, anxiety, loneliness, and personality traits were measured at the pre-assessment time. The outcome measures included positive and negative affect, serenity affect, depressive mood state and anxious mood state; these were measured at two points (i.e., before and after viewing the intervention/control materials). Towards the end, participants received a debriefing form with information about mental health support in case they felt concerned about their mental health. Participants were also invited to leave qualitative feedback regarding their experience of engaging with the well-being tools and soothing images.

### Inclusion and exclusion criteria

Participants were eligible to take part if they met the following criteria: (i) Aged 18 or above; (ii) Self-identified as Chinese; (ii) Currently enrolled as a full-time student in a university; (iv) There was no limitation to participants’ location of study to participate in the research; both domestic and overseas Chinese students could take part.

### Participants

A sample of 275 eligible students provided their consent to participate in the present research. However, only 186 of these students started the experiment, and ultimately 151 participants completed all the measurements (*n* = 76 for the intervention group; *n* = 75 for the control group). Dropout rates at each stage of the study were primarily due to various factors, including scheduling conflicts, personal commitments, and unforeseen circumstances among participants. The final sample comprised 119 females and 32 males (*Mean age* = 22.77; *SD* = 0.23). The majority of the participants studied in China (61.9%) in Arts, Humanities and Social Sciences (59.6%). The largest percentage of courses students were enrolled in an undergraduate programme (47.8%). Further details of the demographic characteristics in the two groups are provided in the Results section below (See Table 1; “Demographics” section). Prior to the beginning of the research, an *a priori* power analysis was conducted through G*Power 3.1 (Faul et al., 2009). Using an α of 0.05, a power level of 0.80 and an assumed medium effect size (0.5), a sample size of 98 participants were required to conduct the planned analyses. Based on this, the current sample was deemed to be adequately powered to test the *a priori* hypotheses.

**Table 1:** Demographic characteristics of the participants (*N* = 151)

Demographic Variable	Frequency	Percentage (%)	Mean age	Std. Deviation
Gender				
Males	32	21.2%		
Females	119	78.8%		
Age			20.82	2.75
Location of Study				
Argentina	1	0.7%		
Australia	2	1.3&		
Bulgaria	1	0.7%		
Chad	1	0.7%		
China	93	61.6%		
Germany	1	0.7%		
Italy	1	0.7%		
Japan	1	0.7%		
United Kingdom	44	29.1%		
USA	6	4.0%		
Subject of Study				
Humanities and Social Sciences	90	59.6%		
Medicine and Veterinary Medicine	10	6.6%		
Science and Engineering	31	20.5%		
Other	20	13.2%		

### Experimental condition and materials

#### Intervention group (Soothe Vision videos)

Participants assigned to the intervention group were asked to watch six videos consecutively. Each video was approximately 2 to 3 min (see links in Appendix 1). Each video consisted of a sequence of eight images accompanied by four literary quotes. Each quote was presented after the display of two images. Thus, the intervention group participants were presented with a total of 48 soothing images and 24 inspirational literary quotes from Harry Potter books. Furthermore, each video has a unique Hobbit-style song specifically produced for the project through commissions with a musician and played alongside the *Project Soothes* images. Similarly, there was a separate piece of tailor-made Hobbit-style music accompanying the presentation of literary quotes. The *Young Citizen Scientist* team, which designed the *Soothe Vision* videos, selected the images, music and literary quotes based on their own soothing experiences in everyday life, supported by research evidence (See Introduction above).

The research team adapted the original English version of the Soothe Vision videos to a Chinese version (i.e., the quotes were translated by authors YY and ZL to Chinese and, where possible, taken from the Chinese version of the Harry Potter books). All Chinese-version videos were made on iMovie by combining translated quotes, pictures, and music from the original designer. The Project Soothe images were selected by the young citizen scientist team from the full collection of 800 images collected by Project Soothe (MacLennan et al., 2023); they were based on five themes: landscapes, water features, trees/flowers, animals and sky (See examples in Appendix 1).

#### Control group (Project Soothe images)

Participants assigned to the control condition were asked to view the same 48 soothing images presented in a set of 6 (i.e., 8 images in each set) used in the intervention group but without the music, quotes and slide show effects. Similar to the intervention group, the control group participants were asked to rate their soothing experience after each set of images before proceeding to the next set of images. The images were rated on a 5-point Likert scale ranging from *‘not soothing at all’* to *‘extremely soothing’*. Participants viewed and rated the soothing feeling at their own pace and time.

### Measures

#### Demographics

Basic demographic information was obtained through several questions, including gender, age, level of education, location of study and the subject of study. Most of the sample comprised females (78.8%) between 18 and 34 years of age, and the participants were primarily from China (61.6%). See Table 1 for the demographic characteristics of the participants.

#### Baseline measures

The following measures were completed at baseline prior to the intervention. The measures used in our study were based on previously standardised instruments with well-established psychometric properties. These measures had been extensively validated in prior research and demonstrated adequate reliability and validity. Therefore, we chose not to reassess face validity in our current study. However, we conducted an examination and provided a report on the reliability of the measures, deeming it essential to validate their consistency within our particular sample. The internal reliability of each measure was checked in the present sample and reported below.

##### Patient health questionnaire (PHQ-9; Kroenke et al., 2002)

The PHQ-9 is a self-administered 9-item Likert scale used to assess the levels of depressive symptoms experienced in the past two weeks. Participants had to rate nine common depressive symptoms according to their experience in the past two weeks from *0 (never) to 3 (nearly every day).* The Chinese version of PHQ-9 has been reported to have good reliability with a Cronbach’s alpha of 0.86 (Wang et al., 2014). Similarly, good internal consistency was found in the present study with a Cronbach’s alpha of 0.86.

##### The generalised anxiety disorder scale-7 (GAD-7; Spitzer et al., 2006)

The GAD is a 7-item, self-rated Likert scale, which was developed as a screening tool to assess the levels of self-reported anxiety symptoms experienced in the past two weeks. Large-scale studies revealed that the GAD measure has a high reliability and procedural validity (Spitzer et al., 2006). The Chinese version of GAD-7 has been tested in Chinese primary care settings and demonstrated an excellent internal consistency with a Cronbach’s alpha of 0.93 (Shih et al., 2022). A similarly good internal consistency reliability was found in the present sample with a Cronbach’s alpha of 0.90.

##### The big five inventory- 10 (BFI-10; Gosling et al., 2003)

This measure was used to assess personality traits based on the big five-factor model of personality. The BFI-10 is a briefer measure adapted from the original Big Five Inventory (BFI; John et al., 1991). The measure comprised of 10 items, with two items per personality trait. The Chinese version of the BFI-10 has been found to be a reliable and valid measure, with Cronbach's alpha ranging from approximately 0.70 to 0.80 (Carciofo et al., 2016). In the present sample, the internal reliability for each trait subscale varied between 0.42 and 0.75 (Agreeableness = 0.42; Conscientiousness = 0.51; Neuroticism = 0.55; Openness = 0.72; Extraversion = 0.75). As per the Introduction, the existing literature evidence suggests that neuroticism and extraversion dimensions are significant predictors of depressive and anxiety symptoms (Yu & Hu, 2022; Karsten et al., 2012; Kotov et al., 2010); therefore, for the purpose of this study, only the extraversion and neuroticism dimensions were included in the analyses. Of note, the low reliability of the neuroticism subscale will be considered in interpreting the findings of the relevant hypothesis.

##### The UCLA loneliness scale (ULS-8; Hays & DiMatteo, 1987)

This self-reported 8-item Likert scale was employed to measure the subjective feeling of loneliness. The scale has demonstrated good psychometric properties in the general population (Hays & DiMatteo, 1987). The Chinese version of the ULS-8 was shown to be reliable among young people, with a Cronbach’s alpha of 0.82 (Xu et al., 2018). The Cronbach’s alpha of ULS-8 was 0.82 on the current sample.

#### Outcome measures

The following outcome measures were completed at the pre-intervention and post-intervention stages (i.e., two-time points). The internal reliability of each measure was checked in the present sample and reported below.

##### International positive and negative affect schedule short form (I-PANAS-SF; Thompson, 2007)

The I-PANAS-SF is a self-report measurement consisting of 10 items, five to measure positive affect (PA) and five to measure negative affect (NA). The participants were asked to rate how they felt at this moment on a 5-point Likert scale from *'not at all'* to *'very much'*. The translated Chinese version has proven reliable in measuring positive and negative mood states, with a Cronbach’s alpha ranging from 0.83 to 0.81 (Liu et al., 2020). The internal consistency reliability in this sample ranged from 0.75 to 0.87.

##### Positive and negative affect schedule-expanded version-serenity subscale (PANAS-X-Serenity; Watson & Clark, 1999)

The serenity subscale, comprising of three items (‘*relaxed, calm, at ease’*) of PANAS-X and an additional item of ‘*soothed’* to capture the intended mood state of feeling soothed (as intended by the design of Project Soothe) were also included. These four items were rated on a 5-point Likert scale ('*not at all'* to'*extremely'*) and summed to give a score for serenity affect in this study. The scale has shown to have adequate psychometric properties in previous research with an internal consistency of α = 0.74 (Watson & Clark, 1999). Cronbach’s alpha of the PANAS-Serenity subscale was 0.85 in the present study.

##### Short form of the profile of mood states– tension-anxiety subscale (POMS-SF-T) and depression-dejection subscale (POMS-SF-D; Shacham, 1983)

The current study employed two subscales of the shortened Profile of Mood States. POMS-SF-T consists of six items monitoring the transient distinct mood states (tension-anxiety), and the POMS-SF-D consists of eight items assessing the transient distinct mood states (depression-dejection). Both subscales used a five-point Likert scale ranging from *0 (not at all) to 4 (extremely)*. The Chinese subscale versions have a high alpha coefficient of 0.75 to 0.89 (Lin et al., 2003). The internal reliability in this sample ranged from 0.93 to 0.95.

### Data analysis

Analyses were conducted using the IBM Statistical Package for Social Science (SPSS) 25, with the significance level set to 0.05 and confidence intervals of 95% in all cases. Preliminary analyses assessed the normal distribution and presence of outliers or extreme values. Based on boxplots, no extreme values were identified. Though several outliers were identified, the 5% trimmed means were close to the original mean, indicating that the outliers' value did not strongly influence the original mean. Further, all data were retained due to the absence of any significant missing data. Moreover, the Normal Q-Q Plots demonstrated reasonably straight lines on all the variables, suggesting normal data distributions of the data. Therefore, parametric tests were carried out.

Additionally, at the initial stage, descriptive statistics of demographic characteristics and baseline and pre-test measures were computed for each group. Pearson Chi-square tests and t-tests were used to examine any pre-existing group differences at baseline. Any group differences in baseline measures were controlled for in secondary sensitivity analyses.

To investigate the mood effects of Soothe Vision and viewing soothing images (hypotheses 1 and 2), five parallel 2 × 2 repeated measures ANOVAs were conducted to test for any changes in positive affect, negative affect, serenity affect, depressive mood states, and anxious mood states, respectively. Each ANOVA contained one between-subject variable (Group: intervention vs control) and one within-subject variable (Time: pre-test vs post-test). Significant effects were further analysed through t-tests. Levene’s F tests were also computed in all cases, and all variables met the assumption of homogeneity of variance. To examine if individual differences in baseline levels of depression, anxiety, personality traits, and loneliness influence the mood effects (hypothesis 3), Pearson’s product-moment correlations were computed to assess the strength and direction of the relationship. Mood changes in positive affect, negative affect, serenity affect, and depressive and anxious mood states were computed by subtracting Pre-intervention, time 1 (T1) scores from Post-Intervention, time 2 (T2) scores.

## Results

### Descriptive analyses

#### Demographics

Table 1 shows the demographic characteristics of the two groups. The majority of the participants in both groups were female. Results of independent t-tests and Chi-square showed no statistically significant differences between the groups in any demographic variables (all *p*’s > 0.12; See Table 2).

**Table 2:** Group differences in demographics (*N* = 151)

Variables	Video (*n* = 76)	Image (n = 75)	t or χ^2^	*p (2-tailed)*
Age *	22.76 (2.70)	22.77 (3.01)	0.02	0.980
Gender- Female	56 (73.7%)	63 (84%)	2.41	0.121
Location of study- China	45 (59.2%)	48 (64%)	9.12	0.426
Subject- Social Science	42 (55.3%)	48 (64%)	2.48	0.478
Education- Undergraduate	40 (52.6%)	39 (52%)	5.90	0.658

*For Age, values represent Means (SDs) and t value; for other variables, values represent Number (Proportions) and Chi Square

#### Pre-intervention group differences

##### Baseline measures & pre-intervention measures

Means, standard deviations and t-tests results on group differences at the baseline and pre-intervention measures are presented in Table 2. Results showed no statistically significant group differences in participants’ baseline levels of depression, anxiety and neuroticism. However, participants in the intervention group scored higher in extraversion and lower in loneliness than the control group. Furthermore, the two groups did not differ significantly in any outcome measures prior to the intervention. See Table 3.

**Table 3:** Group differences in baseline and pre-intervention measures (*N* = 151)

	Total Participants		Intervention (Video)		Control (Image)		Group Differences	
	*M*	*SD*	*M*	*SD*	*M*	*SD*	*t(149)*	*p*
Depression (PHQ-9)	16.83	5.48	16.49	5.35	17.19	5.62	−0.78	0.434
Anxiety (GAD-7)	13.28	5.15	12.83	4.58	13.73	5.66	−1.08	0.282
Loneliness (ULS-8)	17.74	4.48	16.72	4.36	18.79	4.39	−2.90	0.004*
BFI-Extraversion	5.23	1.71	5.54	1.80	4.92	1.57	2.25	0.026*
BFI-Neuroticism	5.91	1.51	5.72	1.55	6.09	1.45	−1.51	0.133
Positive Affect T1	15.75	3.34	15.93	3.36	15.56	3.34	0.69	0.493
Negative Affect T1	10.44	3.38	10.13	3.52	10.76	3.22	−1.14	0.254
Serenity Affect T1	12.60	3.19	12.79	3.32	12.40	3.07	0.75	0.460
Depressive Mood T1	19.01	7.67	18.22	7.99	19.81	7.29	−1.28	0.204
Anxious Mood T1	16.68	6.07	16.17	6.38	17.19	5.74	−1.03	0.306

Asterisks * indicate statistical significance *p* < 0.05; T1 refers to Time 1

### Primary hypotheses 1 and 2

The overall findings showed that all participants, regardless of group, showed a reduction in positive affect, negative affect, depressive mood states and anxious mood states and an increase in serenity affect. However, there was no significant main effect of group or interaction. Detailed results are reported below.

#### Positive affect

**Fig. 1:**
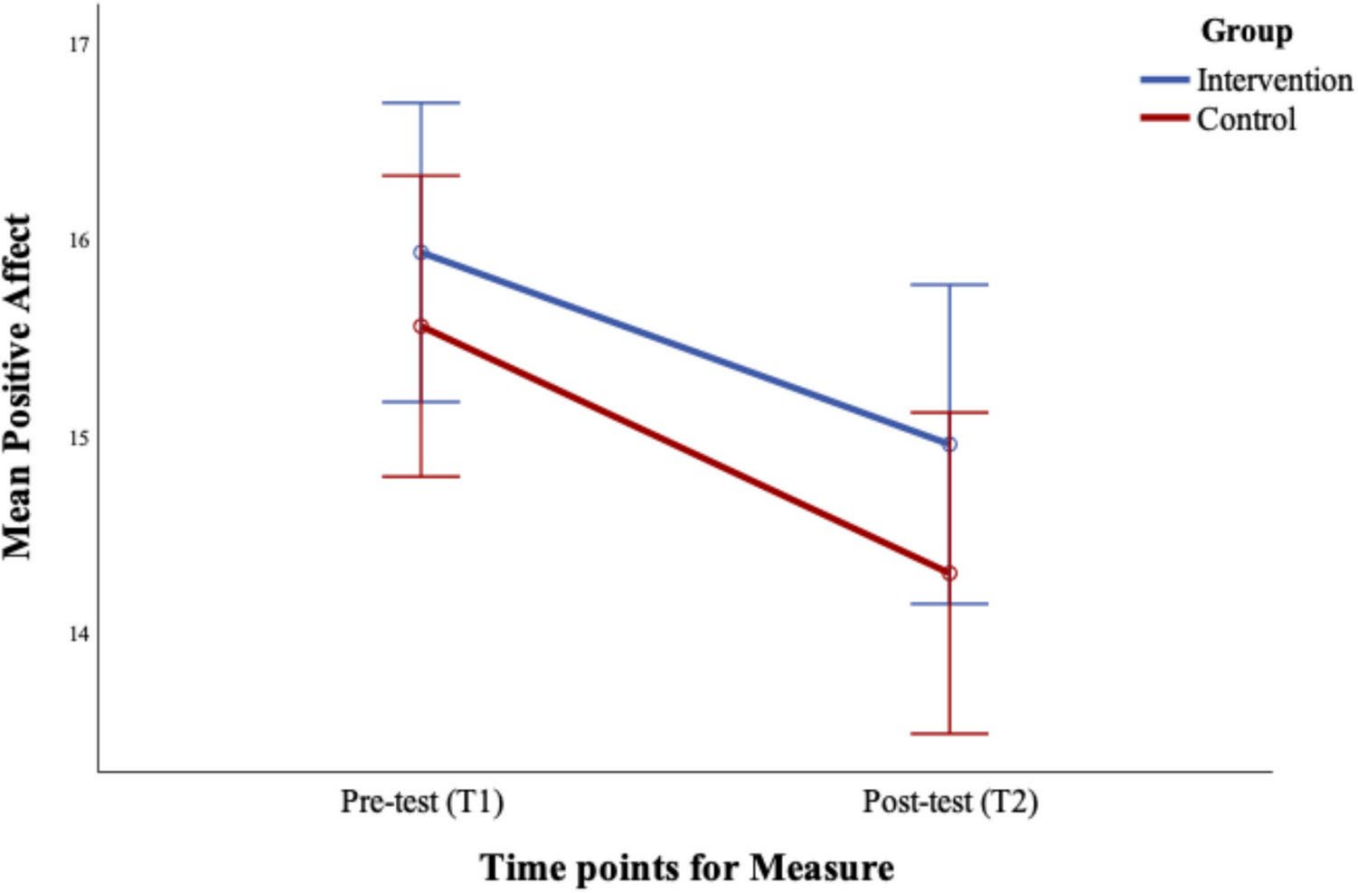
Changes in positive affect pre (T1) to post-intervention (T2). Error bars represent the standard error ± 2SE

There was no significant group difference, F (1, 149) = 1.11, *p* = 0.293, d = 0.007, or Time × Group interaction, F (1, 149) = 0.24, *p* = 0.622, d = 0.002. The main effect of Time was significant, F (1, 149) = 15.46, *p* < 0.001, d = 0.094. Paired sample t-tests confirmed that positive affect reduced over time in both groups; Intervention, t (75) = 2.20, *p* = 0.031, and Control, t (74) = 3.55, *p* = 0.001, indicating that participants showed reduced positive affect regardless of group. See Fig. 1.

#### Negative affect

**Fig. 2:**
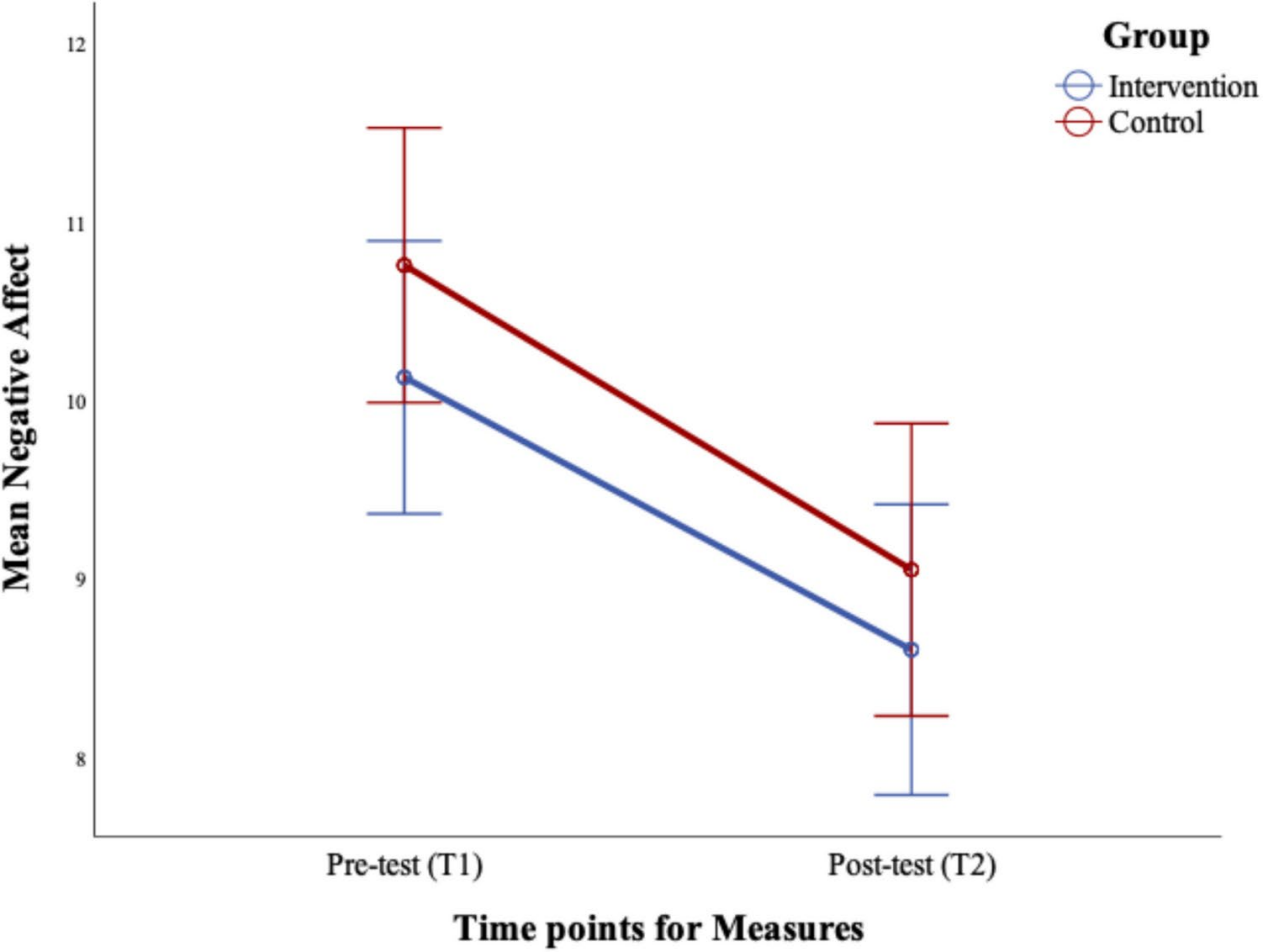
Changes in negative affect pre (Time 1) to post-intervention (T2). Error bars represent standard error ± 2SE

The results demonstrated no significant group difference, *F* (1, 149) = 1.12, *p* = 0.291, *d* = 0.007. or Time × Group interaction, *F* (1,149) = 0.13, *p* = 0.721, *d* = 0.001. Only the main effect of Time was significant,* F* (1, 149) = 41.27, *p* < 0.001, *d* = 0.217, with paired sample t-tests confirming that negative affect reduced over time in both groups; Intervention*, t* (75) = 4.02, *p* < 0.001, and Control, *t* (74) = 5.18, *p* < 0.001. See Fig. 2.

#### Serenity affect

**Fig. 3:**
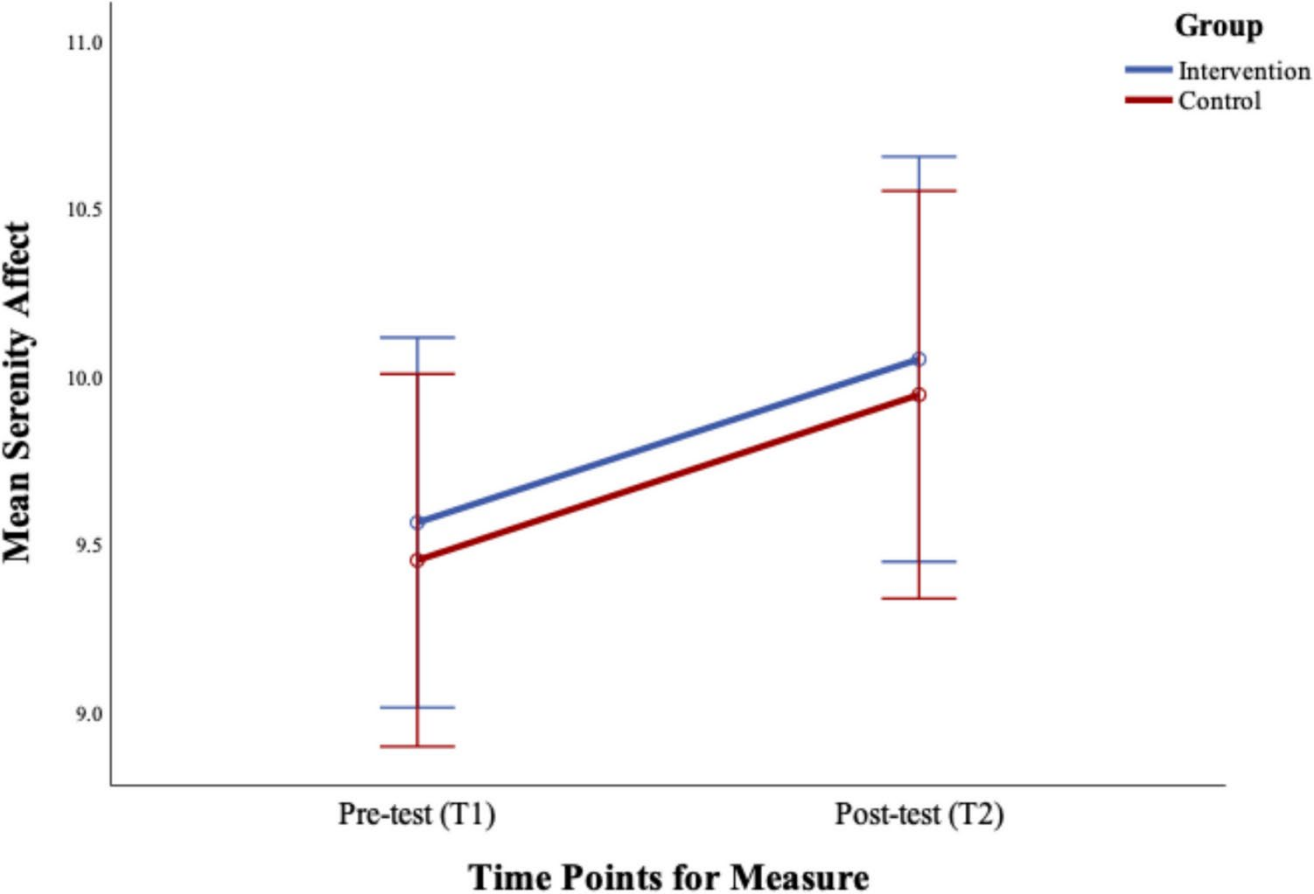
Changes in serenity affect pre (Time 1) to post-intervention (T2). Error bars represent the standard error ± 2SE

The results demonstrated no significant group difference, *F*(1,149) = 0.24, *p* = 0.63 or Time × Group interaction, *F* (1,149) = 0.38*, p* = 0.54. Only the main effect of Time was significant,* F* (1, 149) = 6.08, *p* < 0.001 with paired sample t-tests confirming that the serenity effect increased over time in both groups; Intervention, *t* (75) = −1.58, *p* < 0.001, and Control, *t* (74) = −1.84, *p* < 0.001. See Fig. 3.

#### Depressive mood

**Fig. 4:**
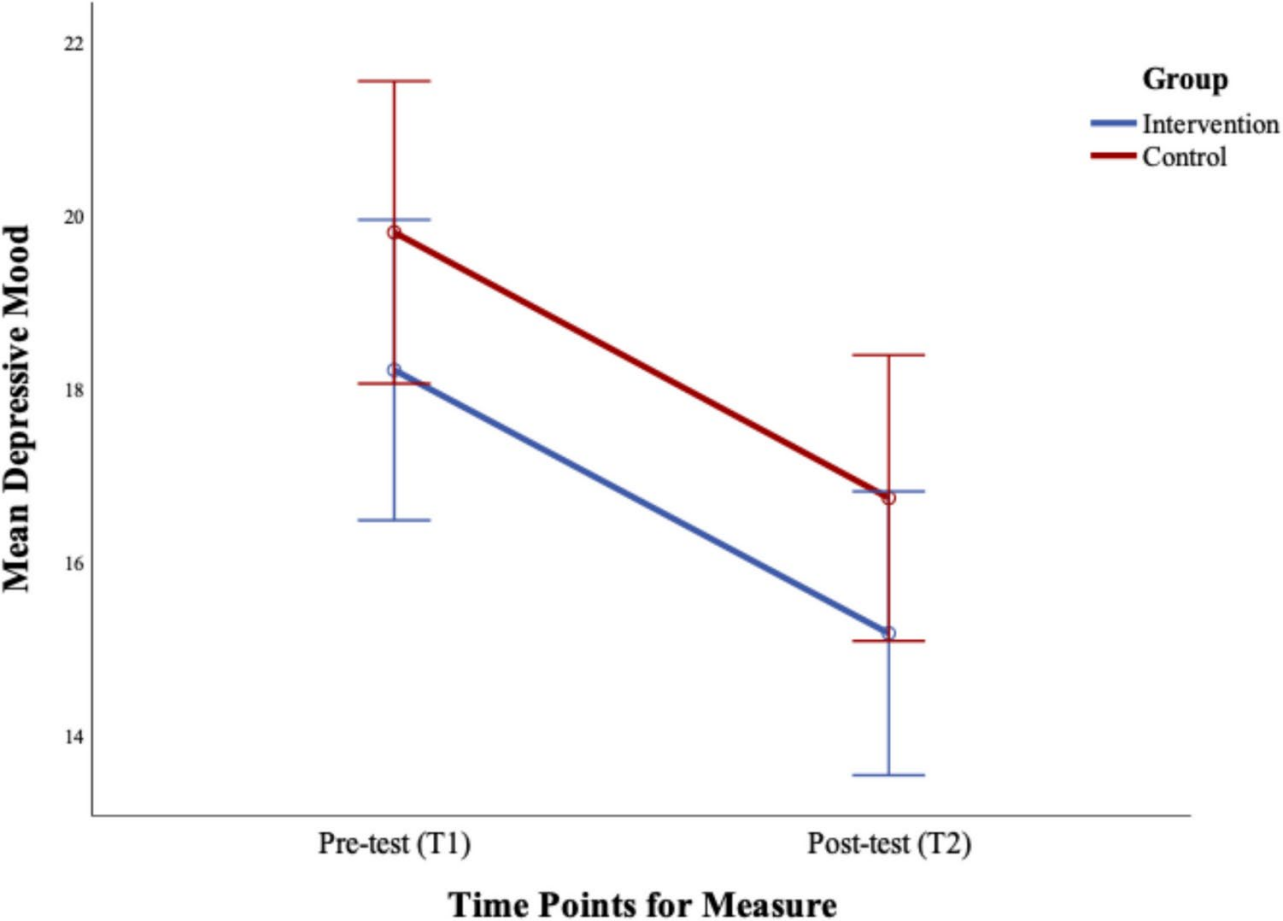
Changes in depressive mood pre (Time 1) to post-intervention (T2). Error bars represent the standard error ± 2SE

The results showed no significant group difference, F (1, 149) = 1.92, *p* = 0.169, d = 0.013 or Time × Group interaction, F (1, 149) = 0.001, *p* = 0.974. Only the main effect of Time was significant, F (1, 149) = 55.37, *p* < 0.001, d = 0.271 with paired sample t-tests confirming that depressed mood states reduced over time in both groups; Intervention, t (75) = 4.87, *p* < 0.001, and Control, t (74) = 5.78, *p* < 0.001. See Fig. 4.

#### Anxious mood states

The results showed no significant Group difference, F (1, 149) = 1.92, *p* = 0.169, d = 0.015 or Time × Group interaction, F (1, 149) = 0.001, *p* = 0.974, d = 0.004. Only the main effect of Time was significant, F (1, 149) = 55.37, *p* < 0.001, d = 0.284 with paired sample t-tests confirming that anxious mood states reduced over time in both groups; Intervention, t (75) = 5.52, *p* < 0.001, and Control, t (74) = 5.39, *p* < 0.001. (See Supplementary Materials, Fig. 5).

#### Sensitivity analyses

Due to the significant group differences in baseline extraversion and loneliness scores, subsequent ANCOVAs were performed with extraversion and loneliness scores separately as covariates. Results remained the same, suggesting that baseline differences in loneliness and extraversion did not influence the mood changes reported above (See. Appendix II for the details of ANCOVA results). Consistent with this, extraversion and loneliness scores were not significantly correlated with any mood changes in this study (See Table 3 below).

### Hypothesis 3

Pearson correlations are presented in Table 4. As expected, baseline depressive symptoms were significantly positively associated with loneliness (r = 0.44, *p* < 0.01) and neuroticism (r = 0.41, *p* < 0.01) and negatively associated with extraversion (r =—0.24, *p* < 0.01). In response to Hypothesis 3, baseline levels of depressive symptoms had significant negative associations with changes in depressive (r =—0.28, *p* < 0.01) and anxious mood states (r =—0.34, *p* < 0.01) and positive associations with serenity change (r = 0.17, *p* < 0.05). These findings indicate that participants with higher levels of depressive symptoms at baseline experienced larger reductions in anxiety and depressive mood states and a larger increase in serenity affect, with effect sizes ranging from small to large.

**Table 4:** Pearson's correlations among the study variables

	1	2	3	4	5	6	7	8	9	10
1. Baseline Depression Symptoms	-									
2. Baseline Anxiety Symptoms	0.77**	-								
3. Baseline Loneliness	0.44**	0.39**	-							
4. Extraversion	−0.24**	−0.22**	−0.42**	-						
5. Neuroticism	0.41**	0.53**	0.49**	−0.34**	-					
6. Changes in Positive Affect	0.11	0.14	0.04	−0.08	0.08	-				
7. Changes in Negative Affect	−0.13	−0.14	−0.01	0.01	−0.16	0.07	-			
8. Change in Serenity Affect	0.17*	0.22**	0.04	−0.09	−0.12	0.19*	−0.22**	-		
9. Changes in Depressive Mood	−0.28**	−0.19*	−0.14	0.08	−0.12	0.03	0.56**	−0.25*	-	
10. Changes in Anxious Mood	−0.34**	−0.24**	−0.22	0.04	−0.17*	−0.04	0.39**	−0.20**	0.72**	-

Mood changes were computed by subtracting time 1 (T1) scores from time 2 (T2) scores; ** Correlation is significant at the 0.01 level (2-tailed); * = Correlation is significant at the 0.05 level (2-tailed)

Similarly, baseline anxiety symptoms were significantly positively associated with loneliness (r = 0.39, *p* < 0.01) and neuroticism (r = 0.53, *p* < 0.01) and negatively associated with extraversion traits (r =—0.22, *p* < 0.01). In response to hypothesis 3, baseline anxiety was shown to be significantly associated with changes in serenity affect (r = 0.22, *p* < 0.01), depressive (r = −0.19, *p* < 0.05), and anxious mood states (r = −0.24, *p* < 0.01), suggesting that higher levels of anxiety symptoms were associated with larger reductions in depressive and anxious mood states and larger increases in serenity affect, with small to medium effect sizes. On the contrary, there were no significant associations between negative and positive affect changes with baseline depressive and anxiety symptoms.

Furthermore, among the baseline loneliness, extraversion, and neuroticism scores, only neuroticism had a significant negative association with changes in anxious mood (r = −0.17, *p* < 0.05), suggesting that participants with a higher neuroticism score reported a larger reduction in anxious mood state, with small effect size. Loneliness and extraversion did not appear to be associated with mood changes.

## Discussion

The present study evaluated the mood effects of a newly developed well-being self-help tool, *Soothe Vision*, among Chinese university students. In support of the first hypothesis, the findings showed that soothing images, with or without the addition of music and literary quotes, helped increase positive mood states and decrease negative mood states. However, inconsistent with hypothesis 2, the combination of music and aspirational quotes with soothing images was not shown to offer any additional mood benefits compared with viewing soothing images alone. Additionally, the present findings demonstrated that participants with higher levels of pre-existing depression and anxiety symptoms reported a larger reduction in depressive and anxious mood states and a larger increase in the serenity affect following exposure to soothing images/videos. Those with a higher neuroticism score also reported experiencing a larger reduction in anxious mood states.

The present findings are consistent with previous studies suggesting that soothing images effectively decrease negative mood states such as negative affect, depressive and anxious mood states (Witten et al., 2022) and enhance well-being, positive mood states, feeling soothed, and psychological health (Witten et al., 2022; Shin & Shin, 2019; Jo et al., 2019). Further, these findings are in line with previous research that demonstrated the beneficial mood effects of nature-related stimuli, including natural scenery, images, and calming environments, on physiological and psychological health (Meidenbauer et al., 2020; Shin et al., 2019; Jo et al., 2019, 2022). Collectively, our research and that of others suggests that exposure to nature-related sensory stimuli, images and sounds (such as those collected in Project Soothe) has the potential to be developed into effective, easily accessible, and simple intervention techniques to boost positive affect and alleviate psychological distress (MacLennan et al., 2023; Witten et al., 2022).

However, findings revealed that adding music and aspirational quotes to the soothing images provided no additional benefits. This is inconsistent with the previous literature that proposed music as an effective psychological treatment to improve mood, stress-coping, emotional health, and quality of life (Hedge, 2014; Chang et al., 2012). The current findings showed that, while soothing images were effective in generating positive mood changes, the addition of music and literary quotes did not provide the hypothesised "boosting effect" of combining stimuli. Similar findings have been reported in recent research with an adult and adolescent sample, where soothing sounds did not boost the effects of images (Witten et al., 2022; Ryynanen et al., 2023). This could occur due to participants experiencing cognitive overload with multiple stimuli (Sweller, 2011). Humans usually have limited capacity for working memory, and increased multisensory input could potentially compromise individuals’ ability to process the stimuli (Phillips-Wren & Adya, 2020). Indeed, based on the qualitative feedback, some participants mentioned that combining music and quotes had made it harder for them to focus on soothing images. Also, a few participants reported that the transition between images and music was somewhat abrupt, attributing to a non-soothing experience.

Another plausible explanation could be variability in individual responses to music. Previous research suggested that the effect of music is highly subjective and dependent on personal preferences (Thomson et al., 2014). Hence, future studies should explore if personalised music may generate better outcomes than pre-selected music. Furthermore, it is important to note that the music and quotes used in the Soothe Vision tool were based on the personal preferences of the citizen scientist team and may not have resonated with all participants. For instance, the choice of Hobbit-style music and Harry Potter quotes might have been perceived as unfamiliar or culturally irrelevant by some participants, particularly Chinese students who might lack interest in or familiarity with the content of these quotes and music. Therefore, future research targeting more culturally diverse music and material would be beneficial to identify factors that may help enhance the mood effects of viewing images.

Further, the present findings showed a decrease in positive affect in both conditions. While these results may appear to be counterintuitive, similar results have, in fact, been found in our recent research, where positive affect decreased after exposure to nature-based sounds and images (Witten et al., 2022; Ryynanen et al., 2023). This could have resulted from participants’ boredom or fatigue due to the relatively long exposure time and multiple stimuli. Another possible explanation is that, consistent with theoretical predictions and the observed increase in the serenity affect, exposure to soothing images helps activate the participants’ soothing system. According to the compassion-focused theory, activating the soothing system would help balance the drive and threat affective systems; in other words, increasing soothing feelings would predict a reduction in the state of alertness or motivation (Gilbert, 2009). As the items in the positive affect scale (i.e., determined, alert, active, attentive, and inspired) tap into the drive system rather than feelings of being soothed, regulating the drive system may result in a reduction of positive affect scores. As such, our findings appear to suggest that the Soothe Vision videos and soothing images effectively activate the participants’ soothing system, which helps down-regulate the drive system.

Furthermore, our findings replicate previous research in suggesting that higher levels of baseline depression and anxiety symptoms were associated with a larger reduction in depressive and anxious mood states and a significant increase in serenity affect (Witten et al., 2022; Ryynanen et al., 2023). Individuals with higher neuroticism also reported a more substantial reduction in anxious mood states after engaging with soothing images and videos. These findings echo previous research in suggesting that individuals with stronger pre-existing negative emotions and vulnerability are likely to benefit more from calming, pleasant and positive stimuli (Shin et al., 2019). This suggests that neurotic traits, characterised by increased sensitivity to stress and negative emotions, may render individuals more responsive to emotional regulation interventions (Yang et al., 2020). In contrast, loneliness and extraversion did not influence mood changes, suggesting that these traits may interact differently with emotional experiences or lack direct relevance in non-social, stimulus-based interventions like Soothe Vision (Bendall et al., 2022). This highlights that the efficacy of soothing stimuli may depend more on baseline emotional vulnerabilities, such as neuroticism, rather than traits governing social behaviours or feelings of disconnection. However, the effect of neuroticism was limited to anxious mood states, warranting cautious interpretation and further investigation into how mood-based interventions can be tailored to address diverse physiological profiles.

These findings are noteworthy as previous research has suggested that individuals with depression and anxiety may be less responsive to positive stimuli, resulting in a reduced ability to benefit from enjoyable or pleasant experiences (Li et al., 2022; Legrand & Price, 2020). This research has highlighted the need to improve emotional reactivity among depressed and anxious individuals to positive stimuli. By showing that soothing images (with or without music/literary quotes) can act as positive stimuli for participants to help induce positive mood changes, particularly for those with higher levels of symptoms and vulnerability (by virtue of higher neuroticism), the present study offers encouraging findings that positive visual stimuli could be developed into therapeutic techniques for this population.

### Clinical implications

Taken together, the present study provides proof-of-concept evidence to suggest that, upon further research and replication, *Project Soothe* images could be developed as a self-help digital well-being tool to help enhance positive mood and reduce psychological distress among university students. Further investigation and replication of these findings are warranted to establish the efficacy and utility of this approach. To facilitate practical implementation, future research could explore specific strategies for integrating soothing images into existing digital mental health platforms or mobile applications. Additionally, targeted interventions could be developed to tailor the use of these images to individual needs, such as through personalised recommendations based on user preferences and mental health profiles.

Furthermore, recent research has successfully demonstrated the effective use of virtual reality (VR) technology with the combination of artwork and psychotherapy to improve positive affect, mood disorders and emotional dysfunction (Totterdell & Poerio, 2021; Fodor et al., 2018). Moreover, the study underscores the importance of promoting self-help and digital well-being tools to address the challenges of long waiting times for clinical referrals and reduce the stigma associated with seeking mental health support among young people. These tools offer accessible and cost-effective alternatives that can complement traditional psychotherapeutic approaches. By empowering individuals with practical techniques for managing their mental well-being, such interventions have the potential to enhance resilience and foster positive mental health outcomes in diverse populations. Further research could focus on evaluating the effectiveness of specific implementation strategies, such as peer-led support groups or digital mental health literacy programs, in enhancing the reach and impact of these interventions within university settings.

### Limitations

Firstly, the sample was predominately Chinese Undergraduate students with an over-representation of females, thus limiting the generalisability of the present findings to the wider student population. Additionally, the research was delivered through online settings that may have introduced confounding factors such as distractors, poor video or image quality, internet speed, and the experiment environment. Further, the neuroticism subscale of the Big Five Personality Inventory used in the current research was shown to have inadequate reliability; results regarding neuroticism should, therefore, be treated with caution until they are replicated in the future. Additionally, the present research involved Hobbit-style music and Harry Potter quotes based on the personal preferences of the co-production team of young people. Future research should consider more culturally diverse music and quotes to cater for individual differences across a wide variety of young people. Developing a way to allow users to personalise the tools may also be beneficial. Besides, we acknowledge that the participants may experience fatigue due to the number of questionnaires used in the present research. Also, the present study did not include a blank control group, which could have provided additional insights into the intervention effects by comparing them against a completely inactive control condition. Future research could explore including a blank control group to provide a more comprehensive understanding of the effects of the intervention.

Moreover, this study was not pre-registered as an intervention trial. However, for future studies of a similar nature, pre-registration is recommended as a best practice in scientific research to foster greater transparency and mitigate potential bias. Finally, due to the absence of a follow-up period, the present research could not observe any long-term effects of the intervention. This limitation emphasises the necessity for future research to incorporate follow-up assessments, enabling exploration of the sustainability and potential long-term impacts of the intervention over extended periods.

### Conclusion

Albeit with methodological limitations, this study provided preliminary evidence in suggesting that soothing images, presented alone or with music and literary quotes, are potentially effective in improving positive mood states and decreasing negative mood states in university students, especially in those with higher levels of depressive and anxiety symptoms as well as those with higher scores on the personality risk factor of depression. While these findings should be interpreted with caution until they are further replicated and extended by larger scale research studies, they are nonetheless encouraging. More generally, considering the global impact of mental health issues among young people, digital well-being tools offer much potential to be developed as easily accessible, cost-effective, and far-reaching interventions.

## Supplementary Information

Below is the link to the electronic supplementary material.

Supplementary file1 (DOCX 12517 KB)

Supplementary file2 (DOCX 16 KB)

## Data Availability

The data that support the findings of this study are available on request from the corresponding author. The data are not publicly available due to privacy or ethical restrictions.
