# Supplementary material for: Effects of the Soothe Vision well-being tool on university students’ mood: a pilot study
Source: Curr Psychol. 2025 Mar 31;44(10):9112–28. doi: 10.1007/s12144-025-07649-7 (PMC12144047; doi:10.1007/s12144-025-07649-7)
Supplement: Supplementary file 1 — Supplementary file1 (DOCX 12517 KB) [file 12144_2025_7649_MOESM1_ESM.docx]

**Supplementary Materials**

**Appendix - I**


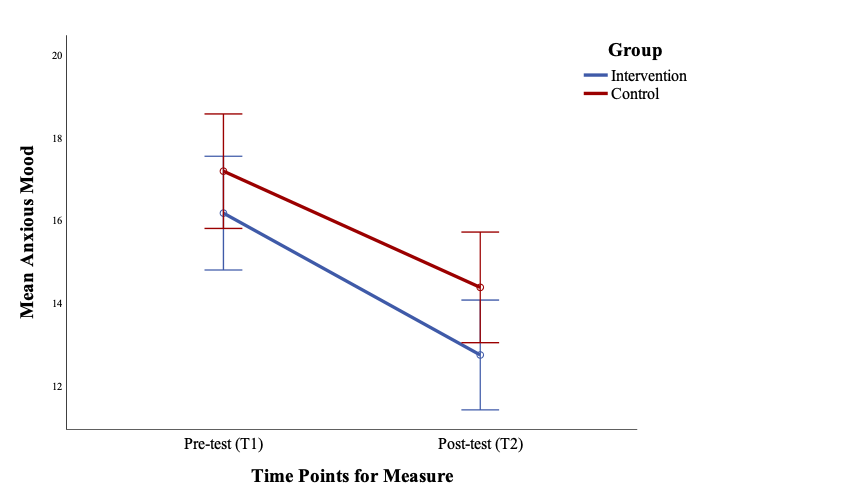


**Figure 5. Changes in Anxious Mood Pre (Time 1) to Post-Intervention**

**(T2). Error Bars represent the Standard Error +/- 2SE**

**Appendix 1**

**Links for *Soothe Vision* Videos**

Video 1: <https://vimeo.com/740502357>

Video 2: <https://vimeo.com/740501125>

Video 3: <https://vimeo.com/740502838>

Video 4: <https://vimeo.com/740501484>

Video 5: <https://vimeo.com/740500789>

Video 6: <https://vimeo.com/740501961>

**Set 1 of Soothing Images**


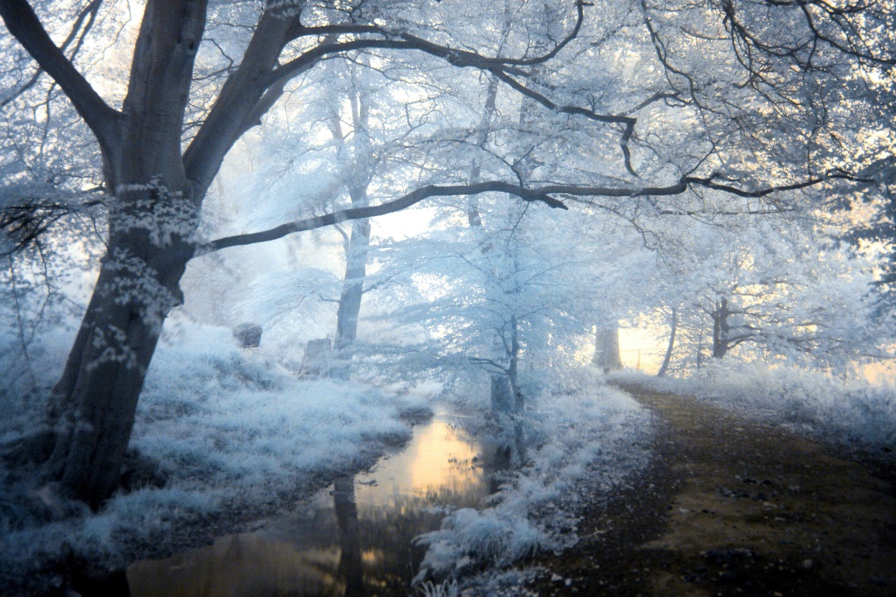


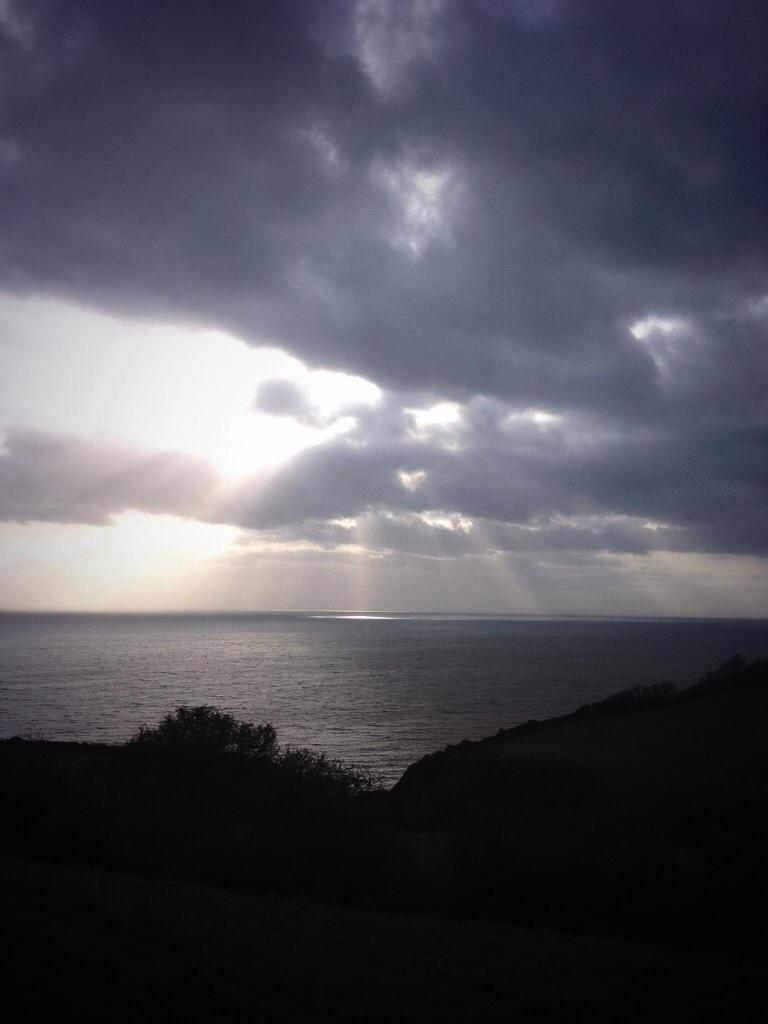


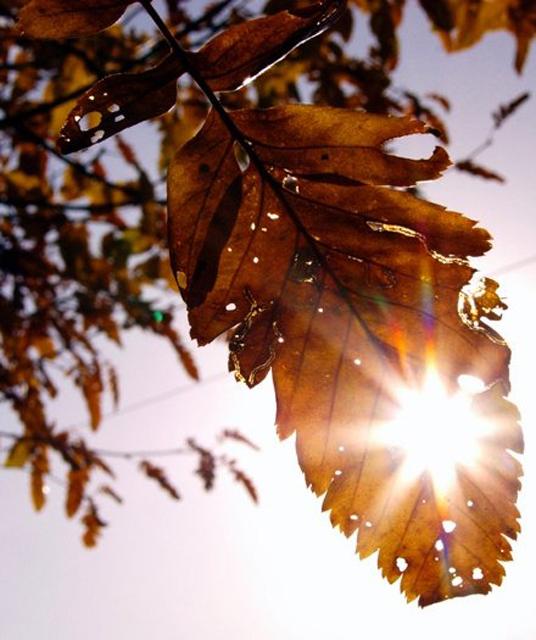


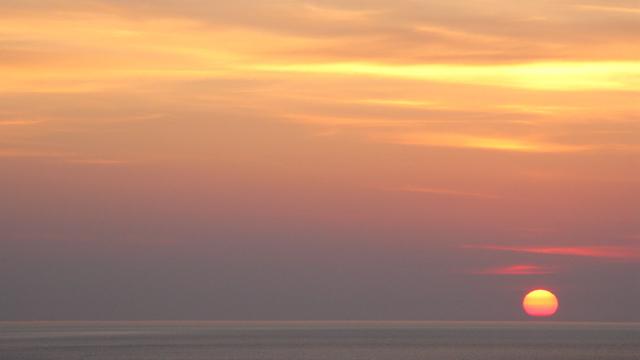


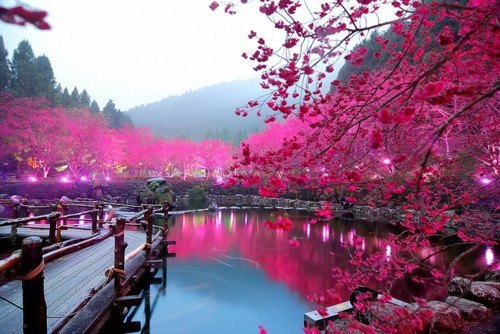


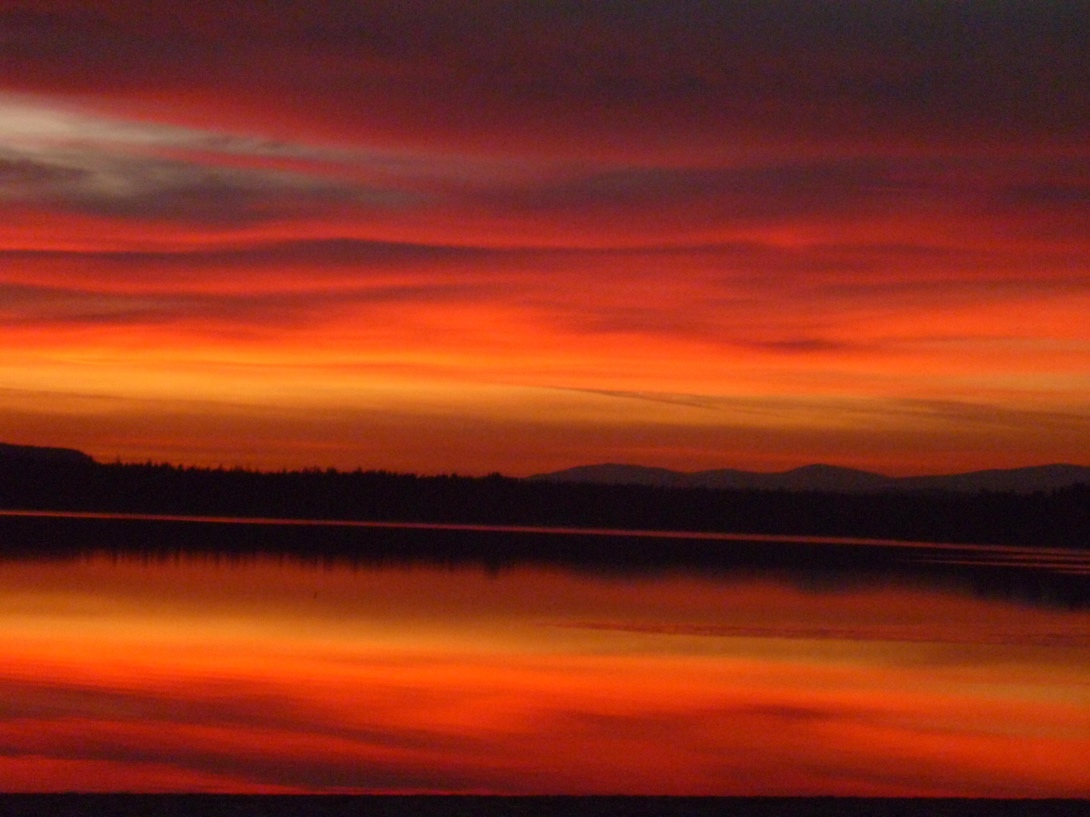


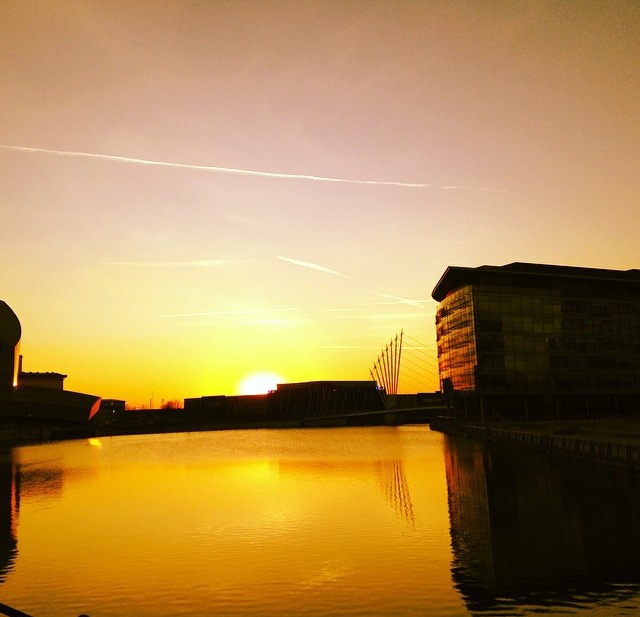


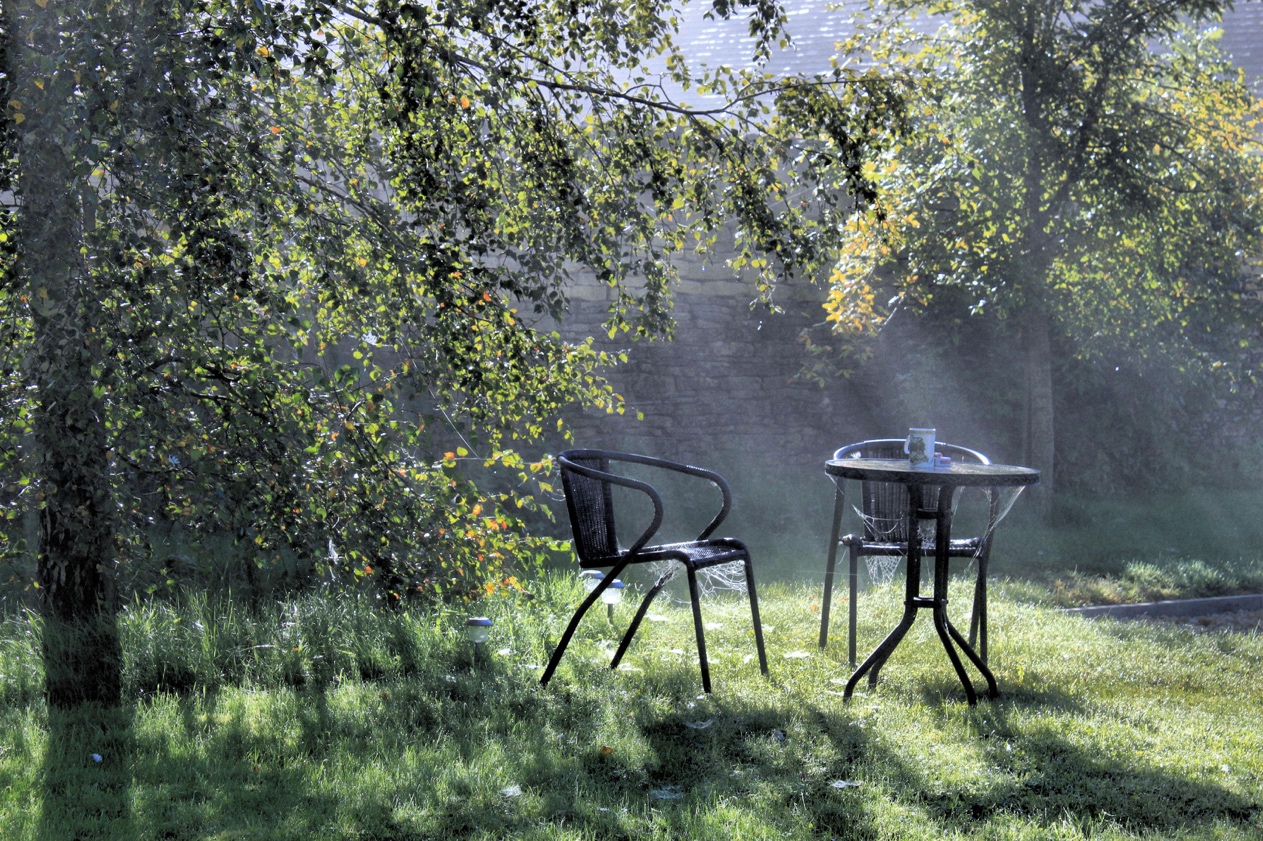


**Set 2 of Soothing Images**


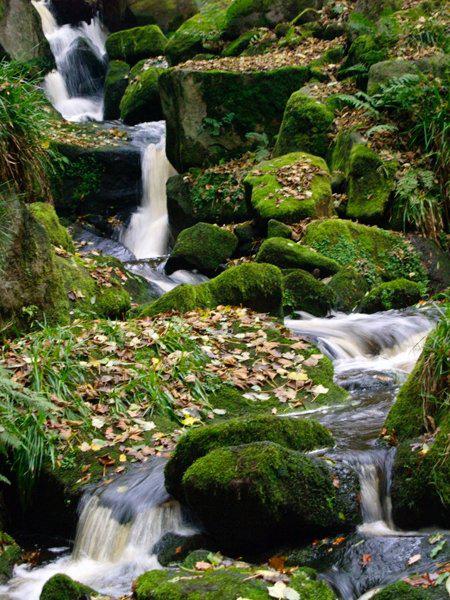


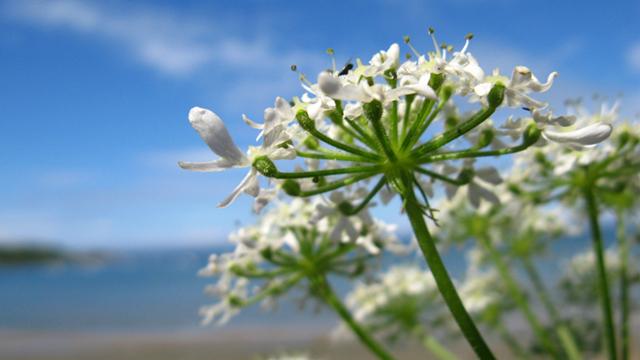


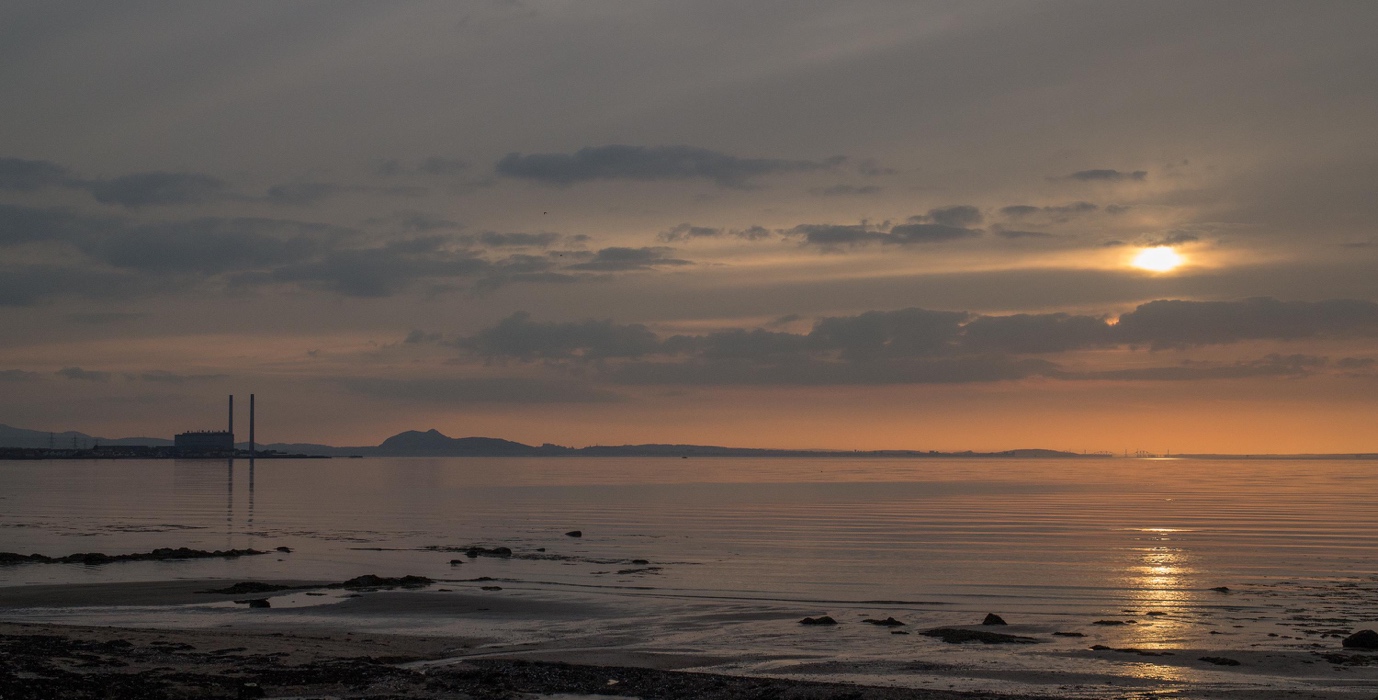


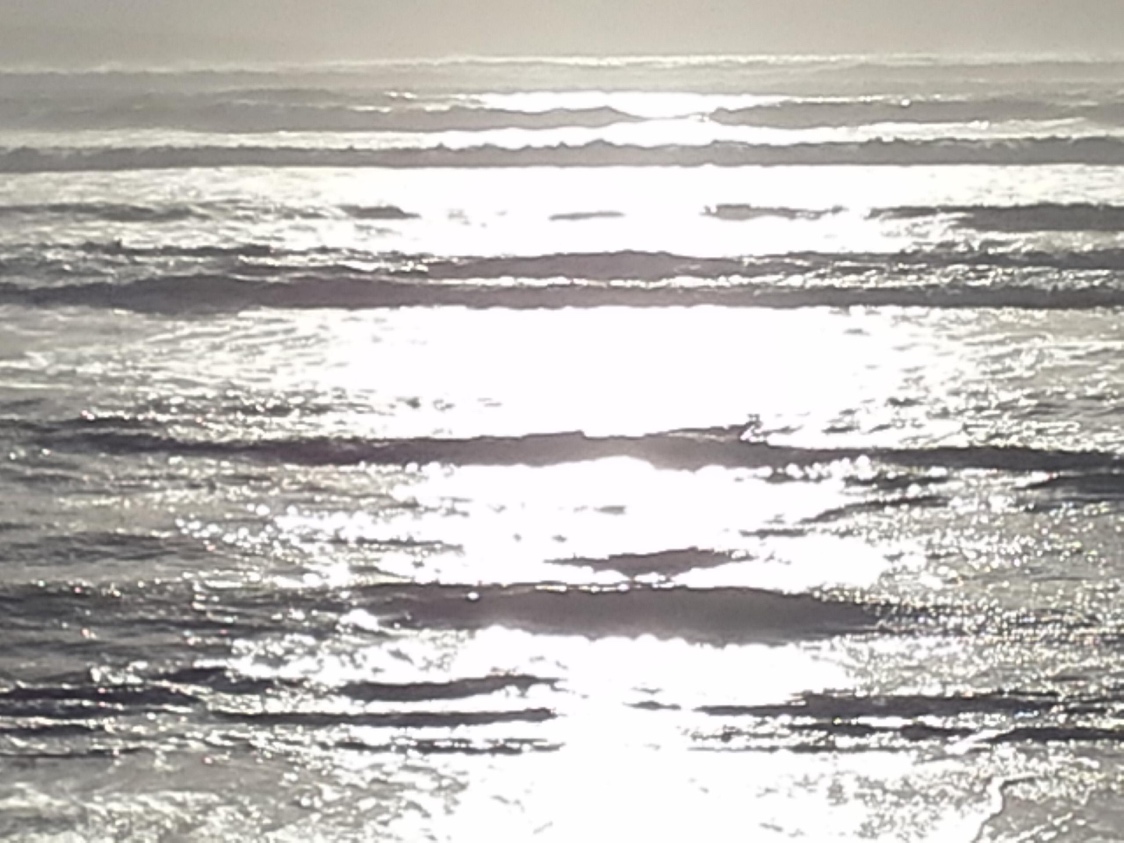


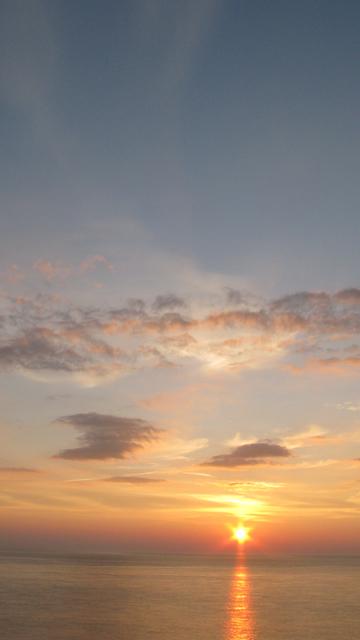


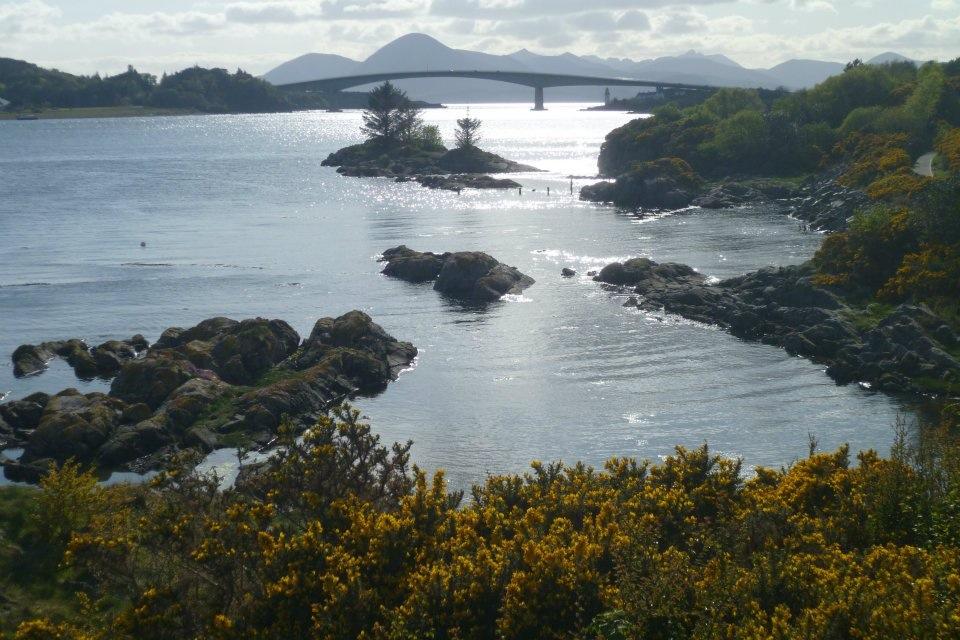


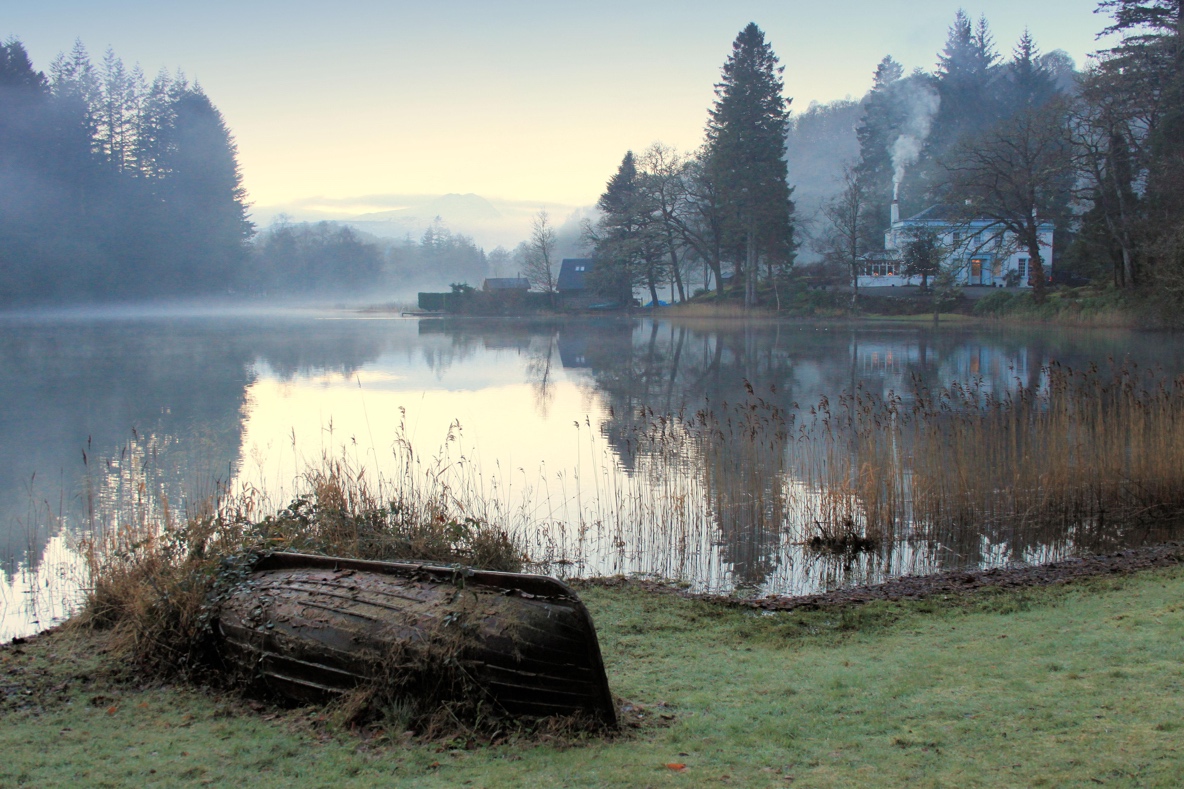


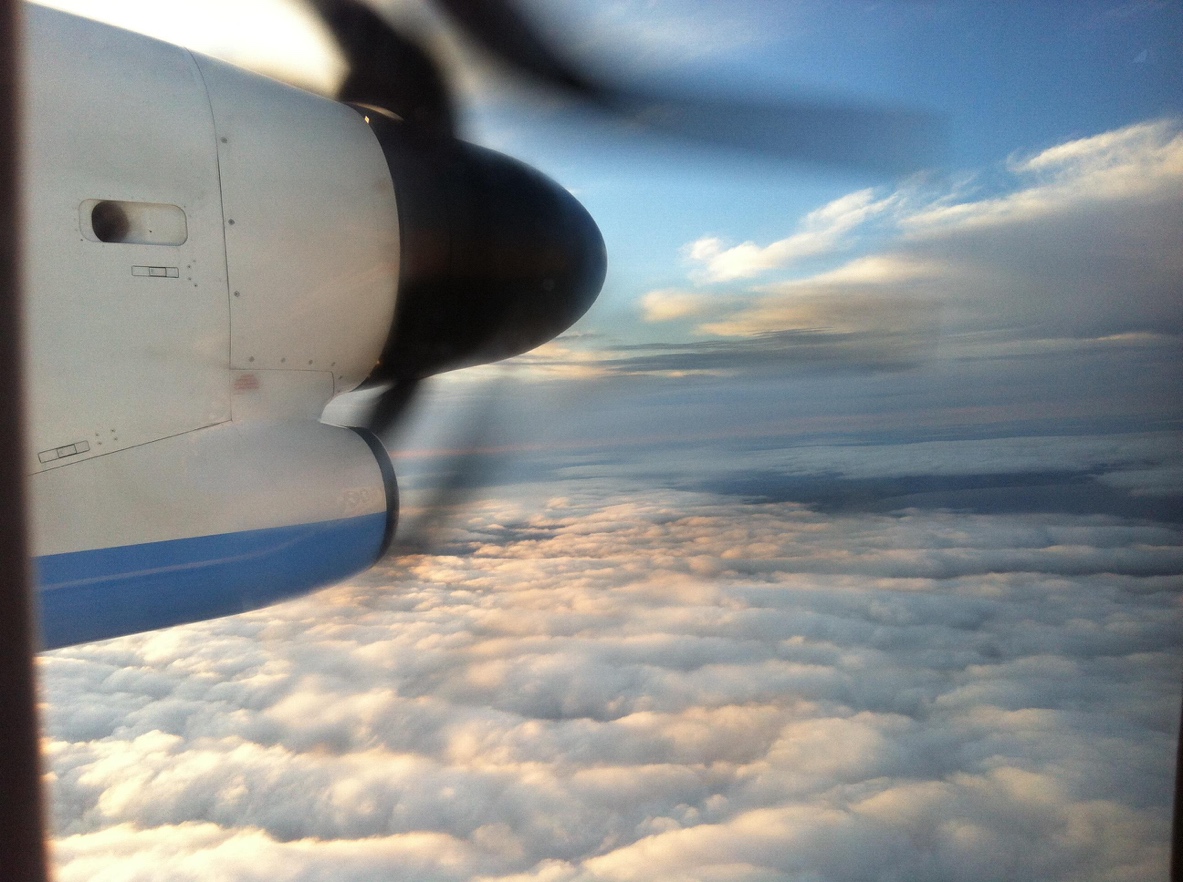


**Set 3 of Soothing Images**


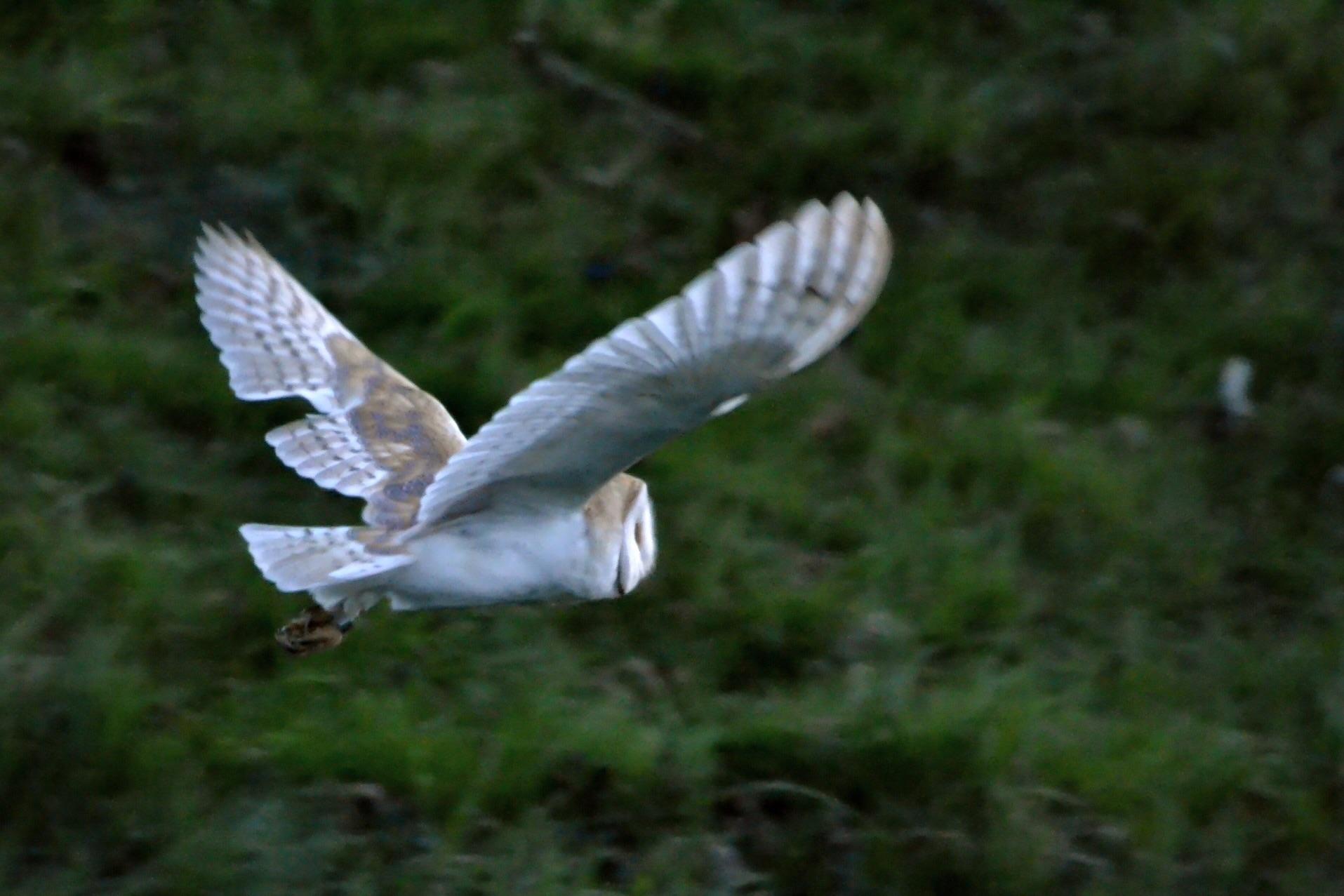


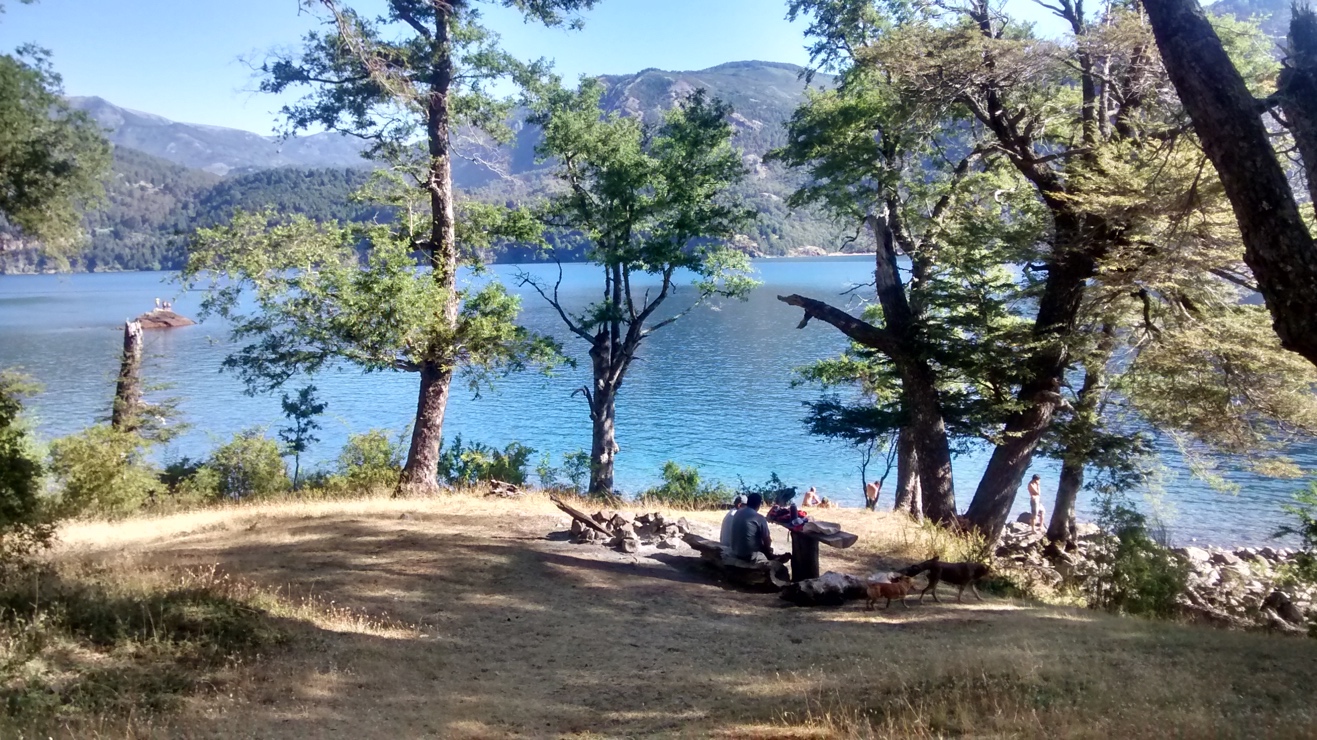


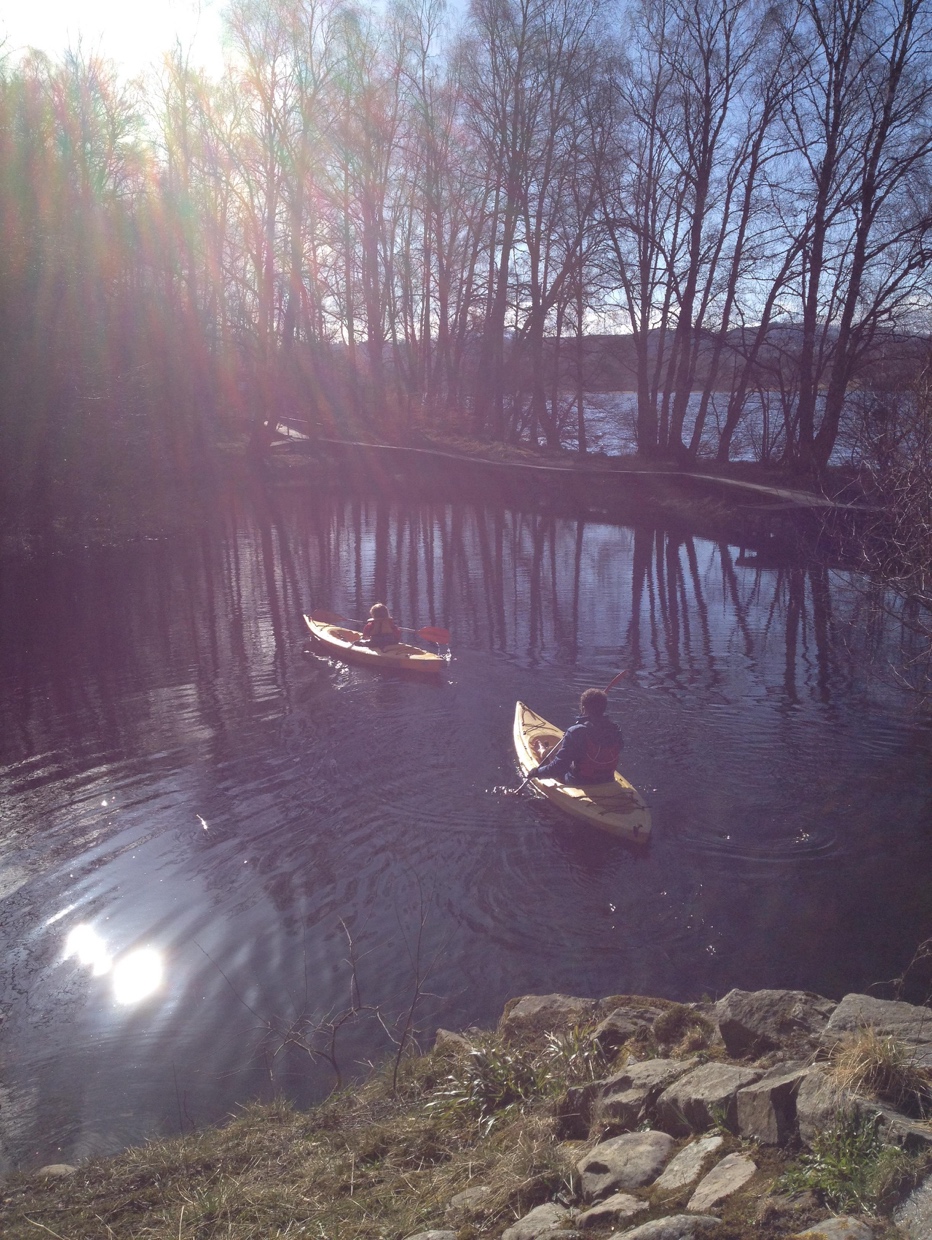


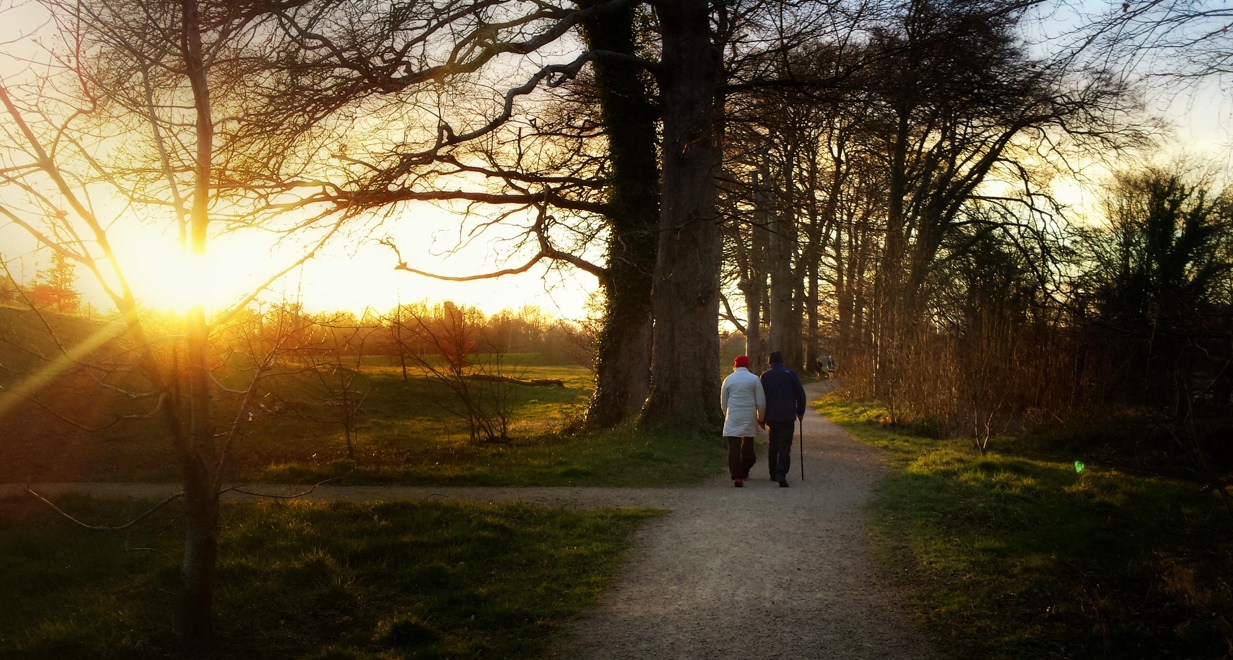


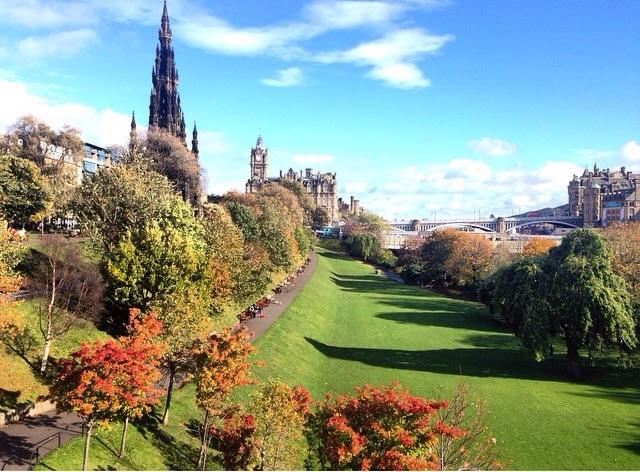


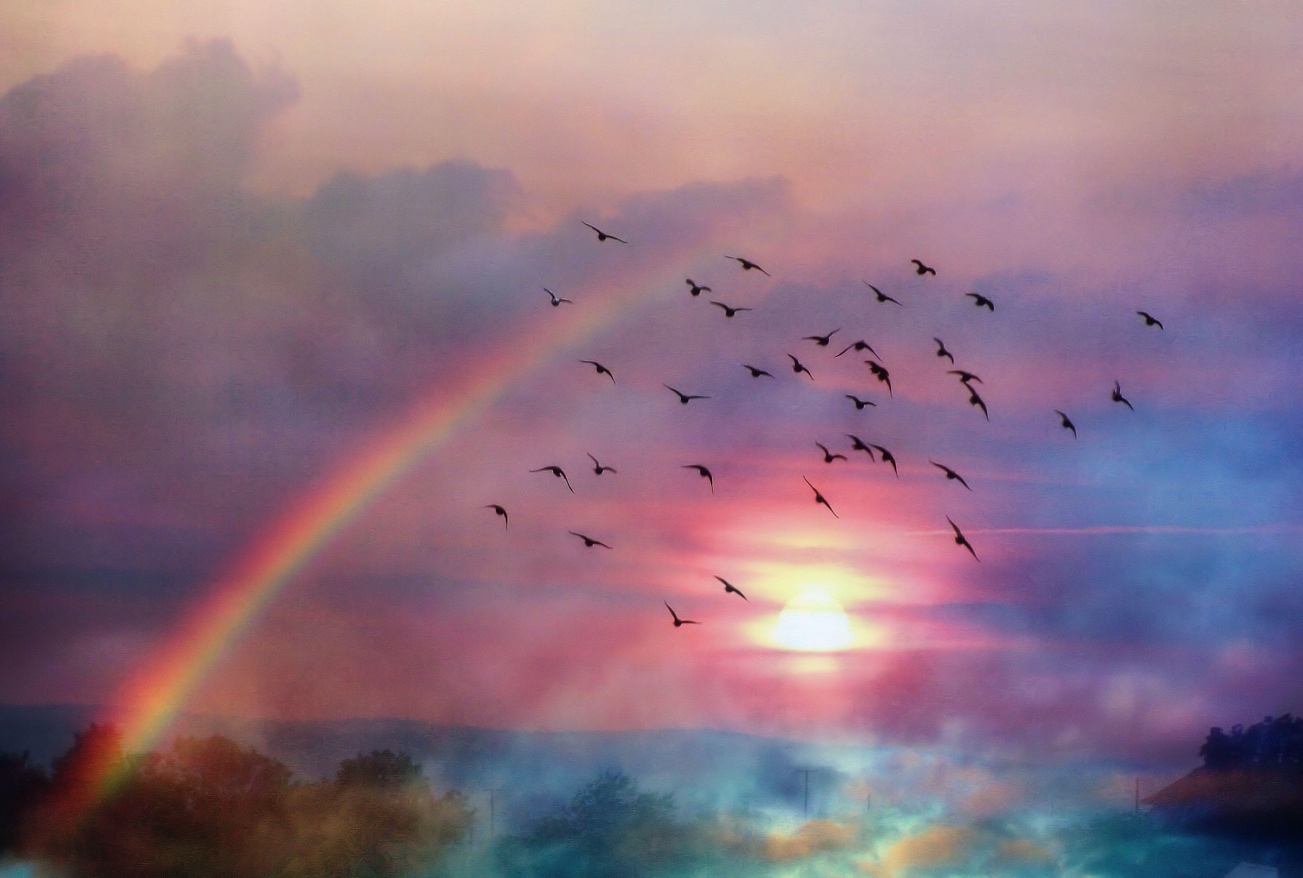


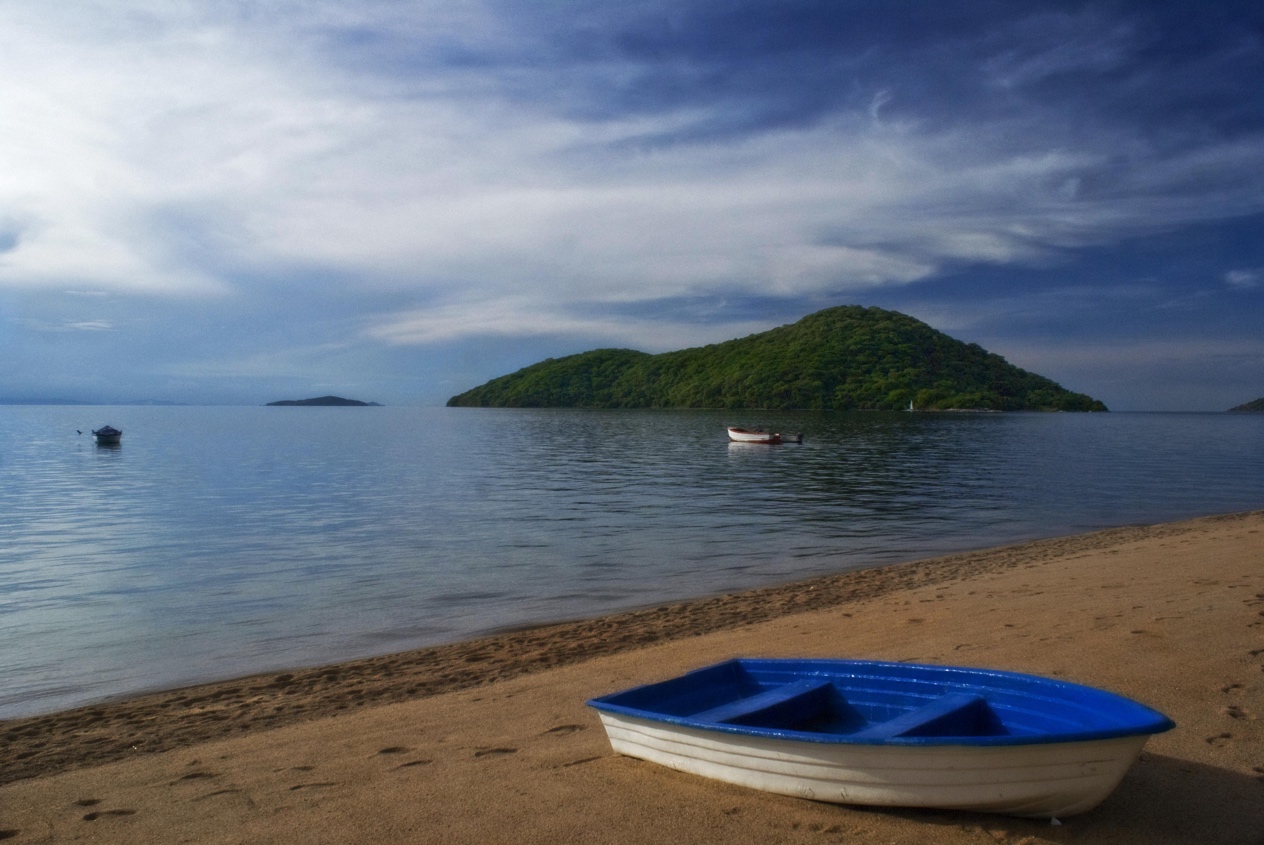


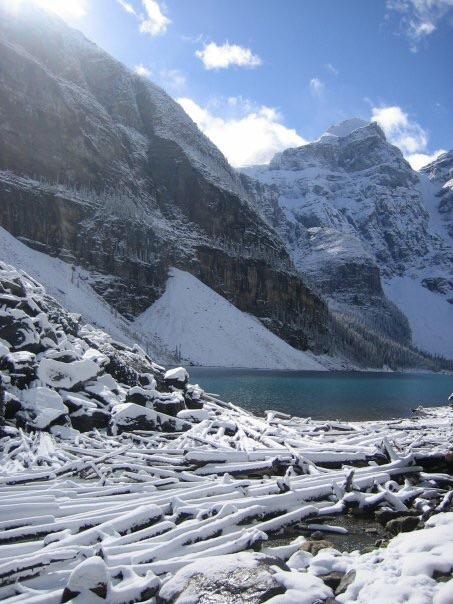


**Set 4 of Soothing Images**


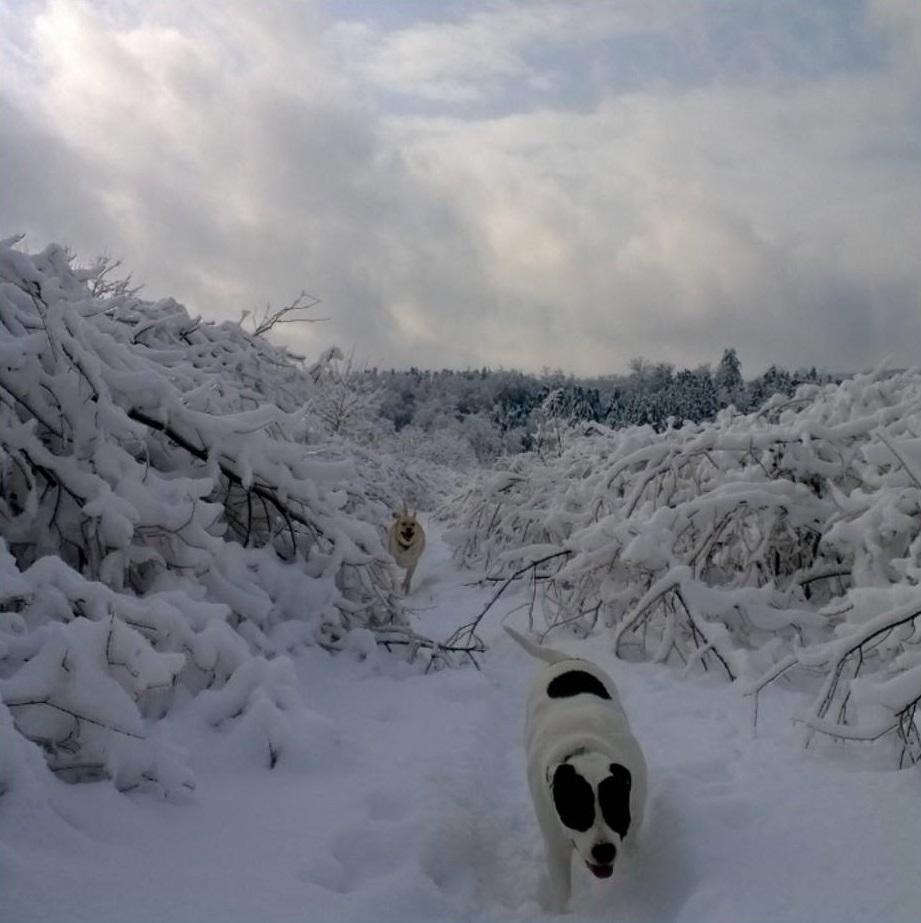


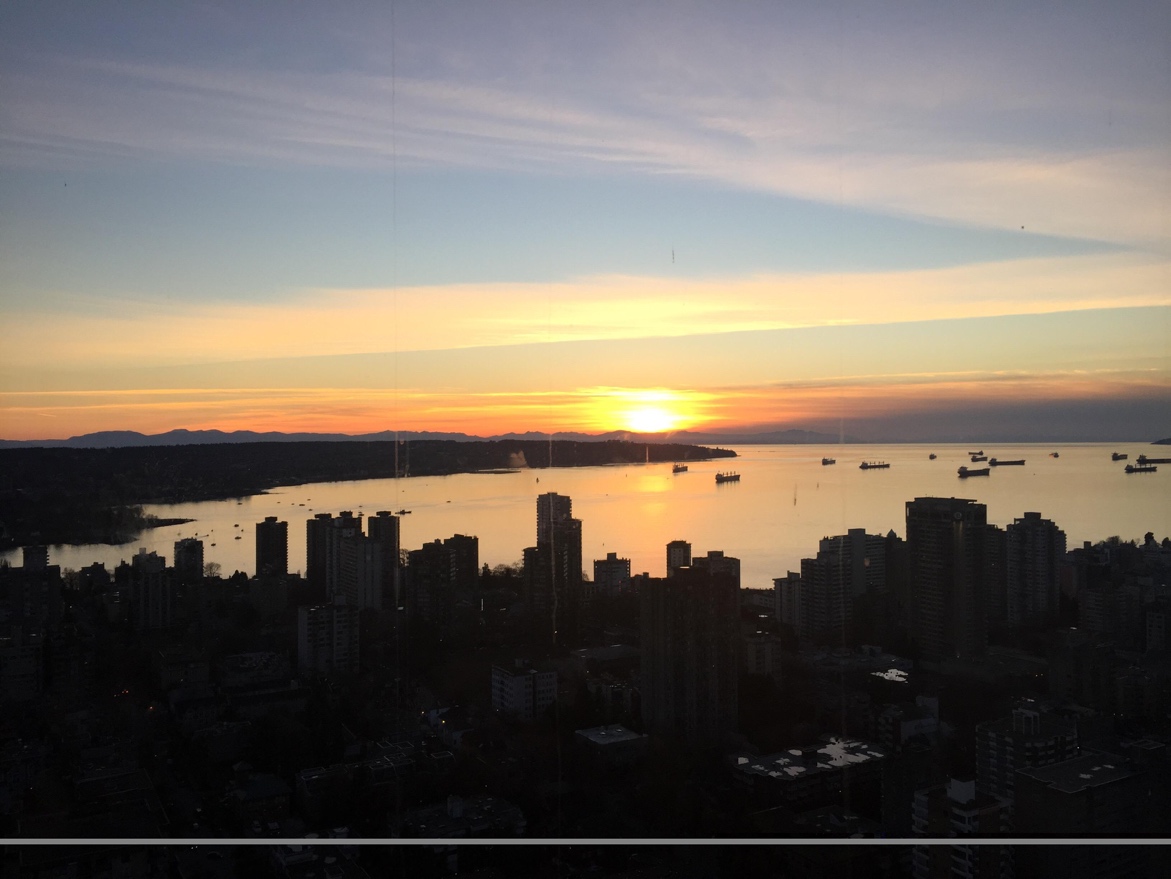


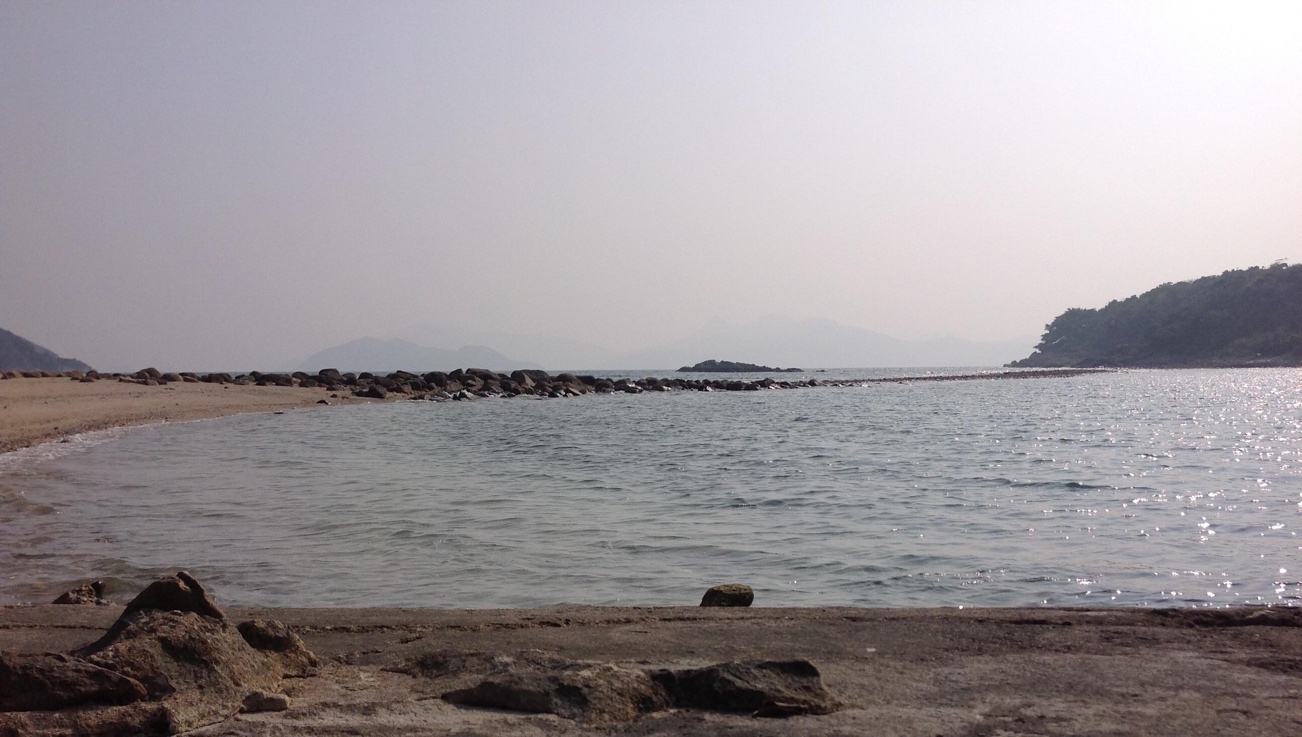


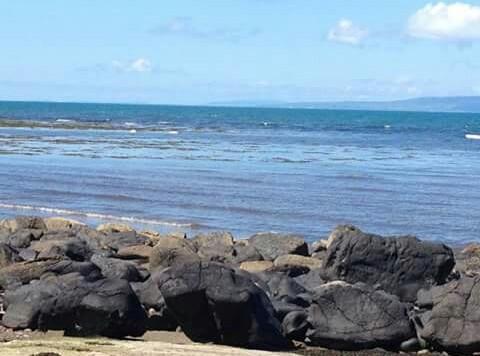


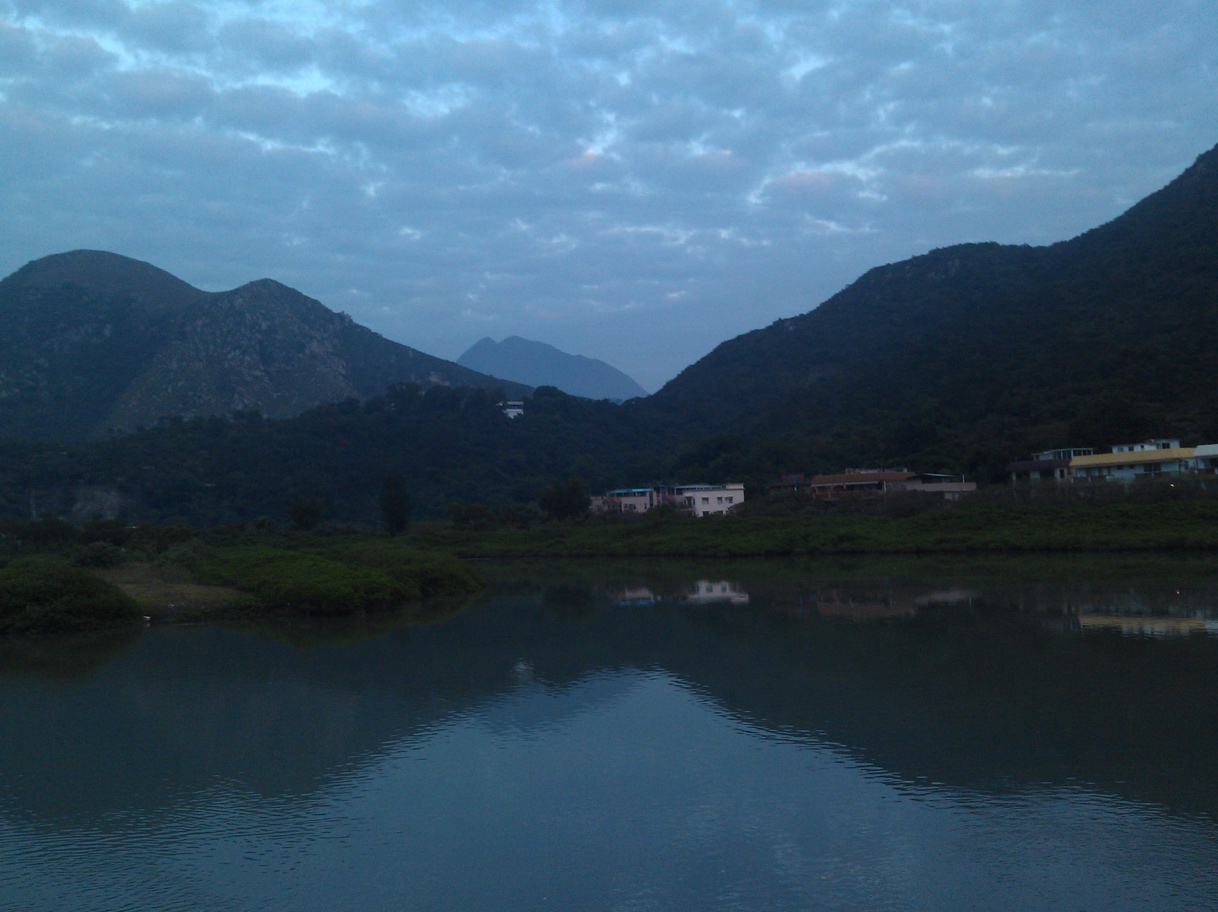


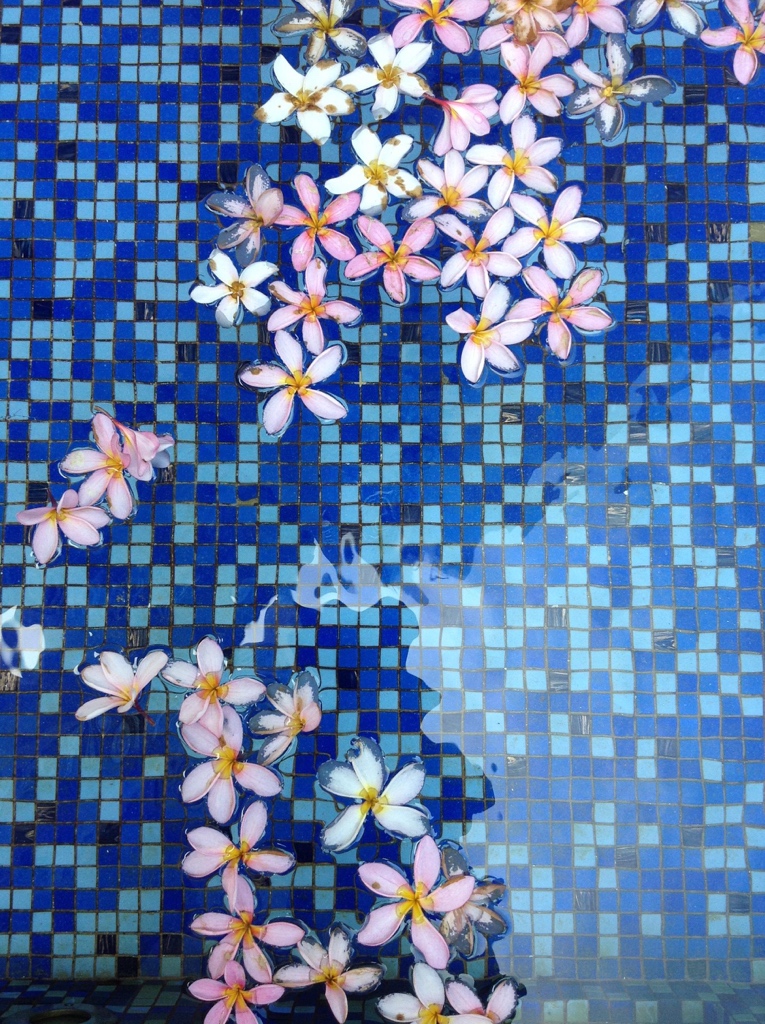


**Set 5 of Soothing Images**


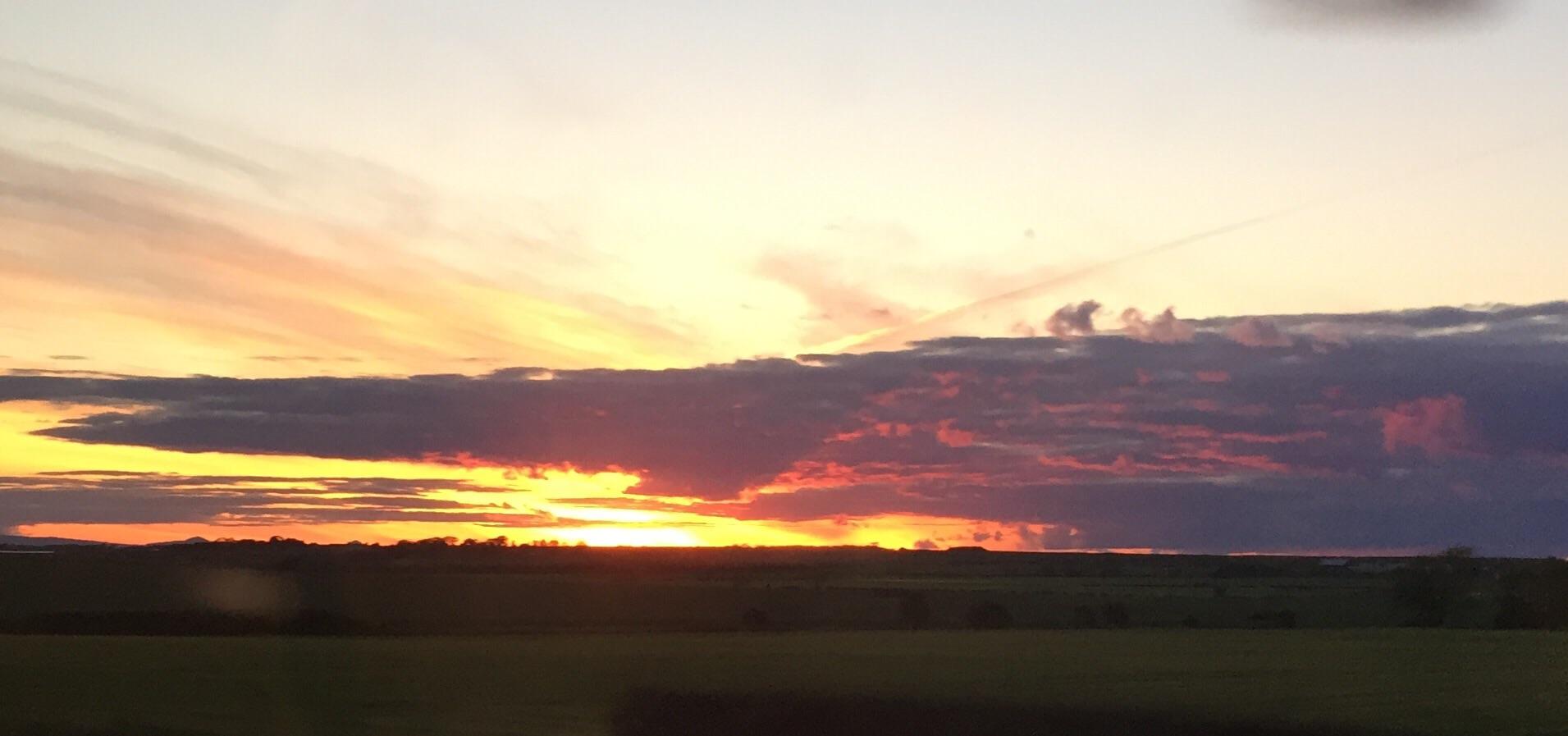


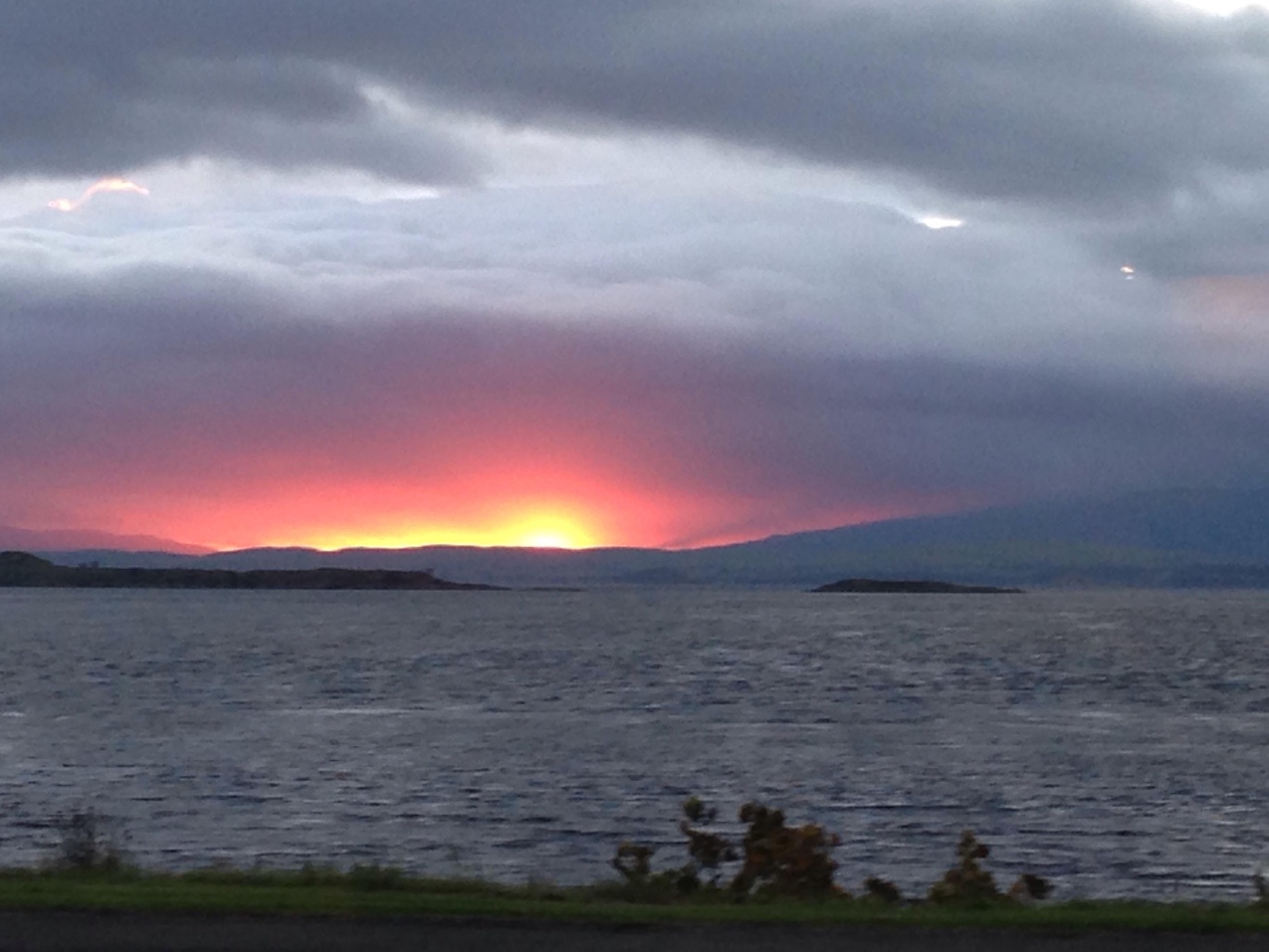


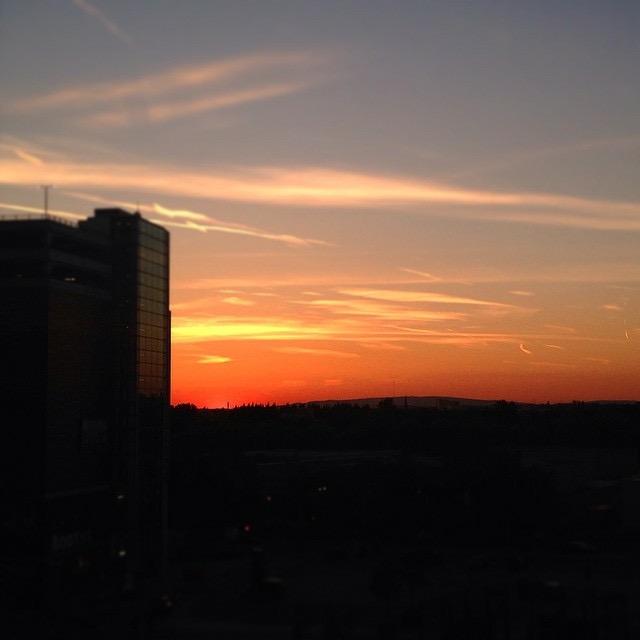


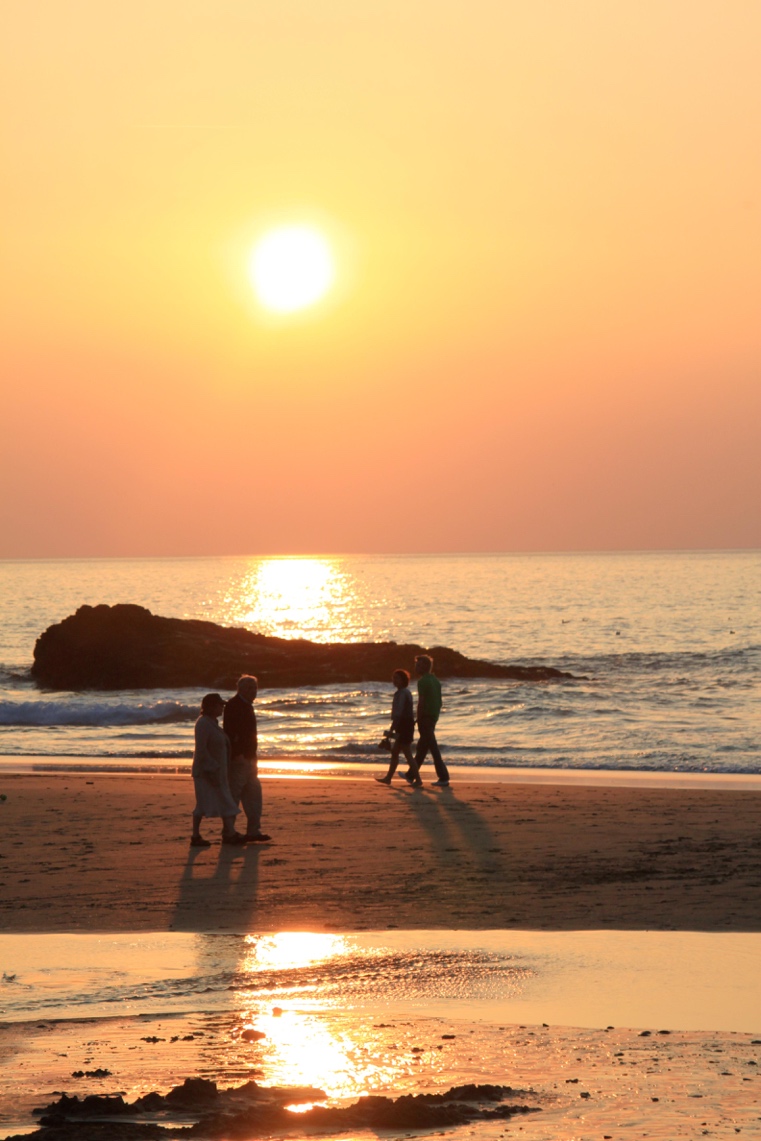


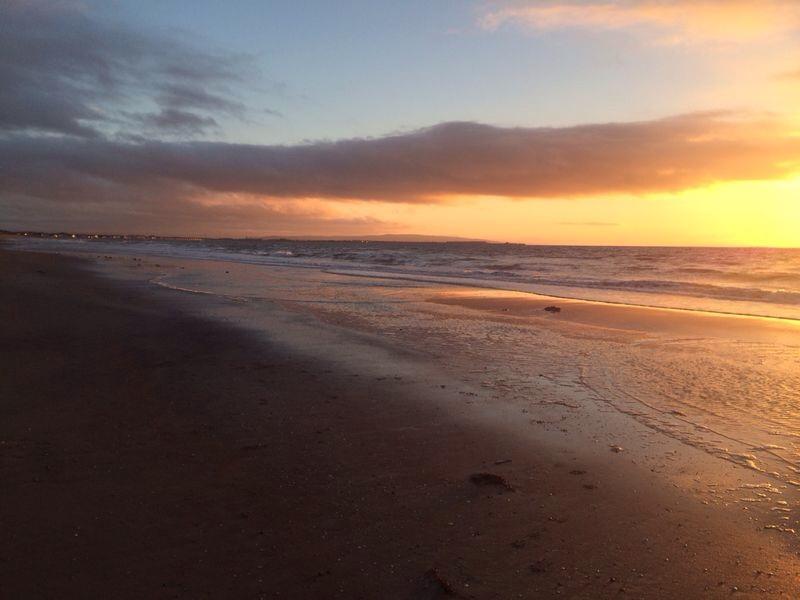


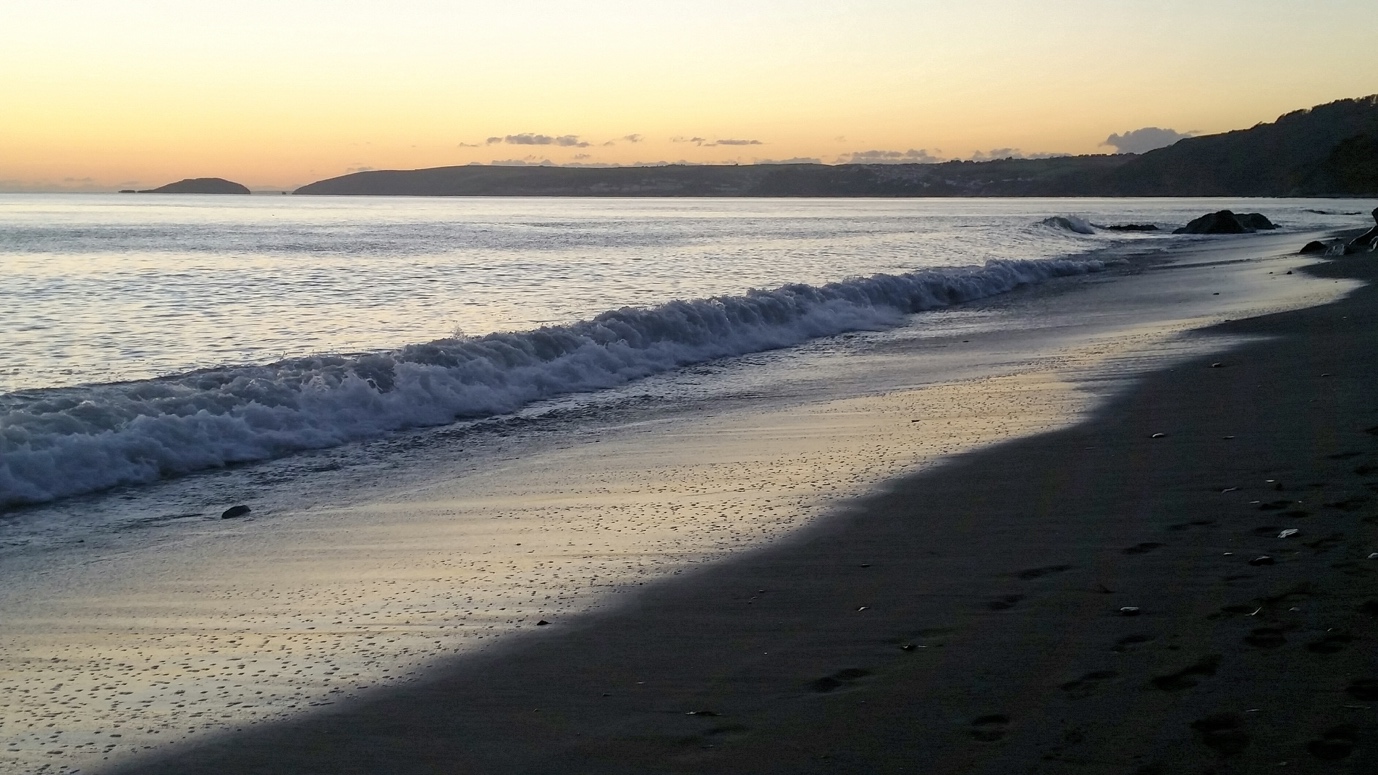


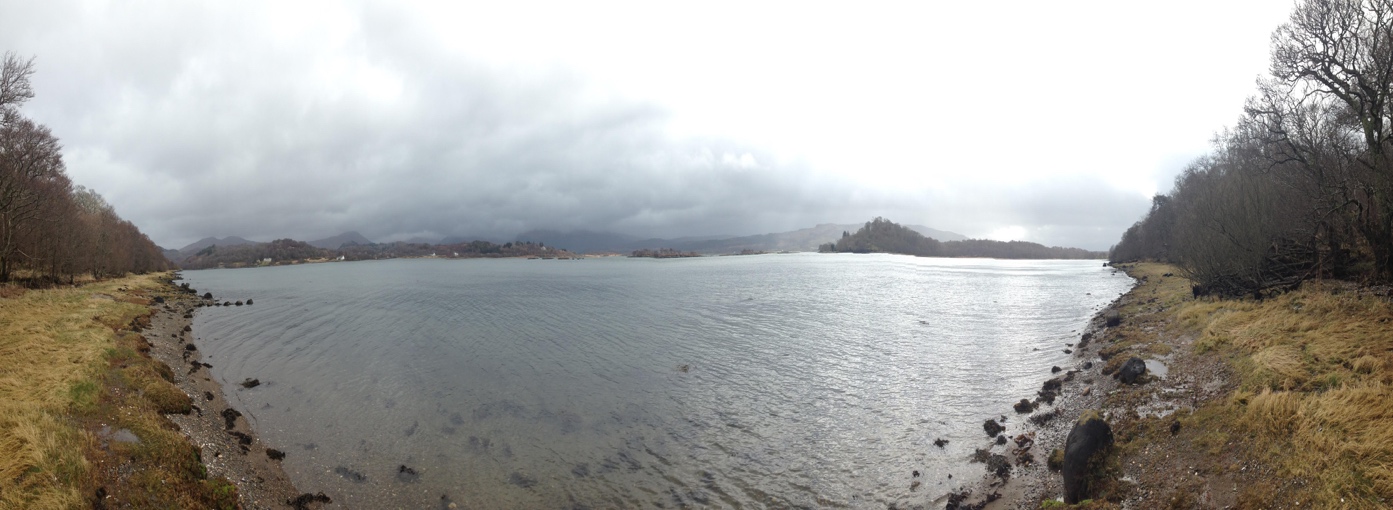


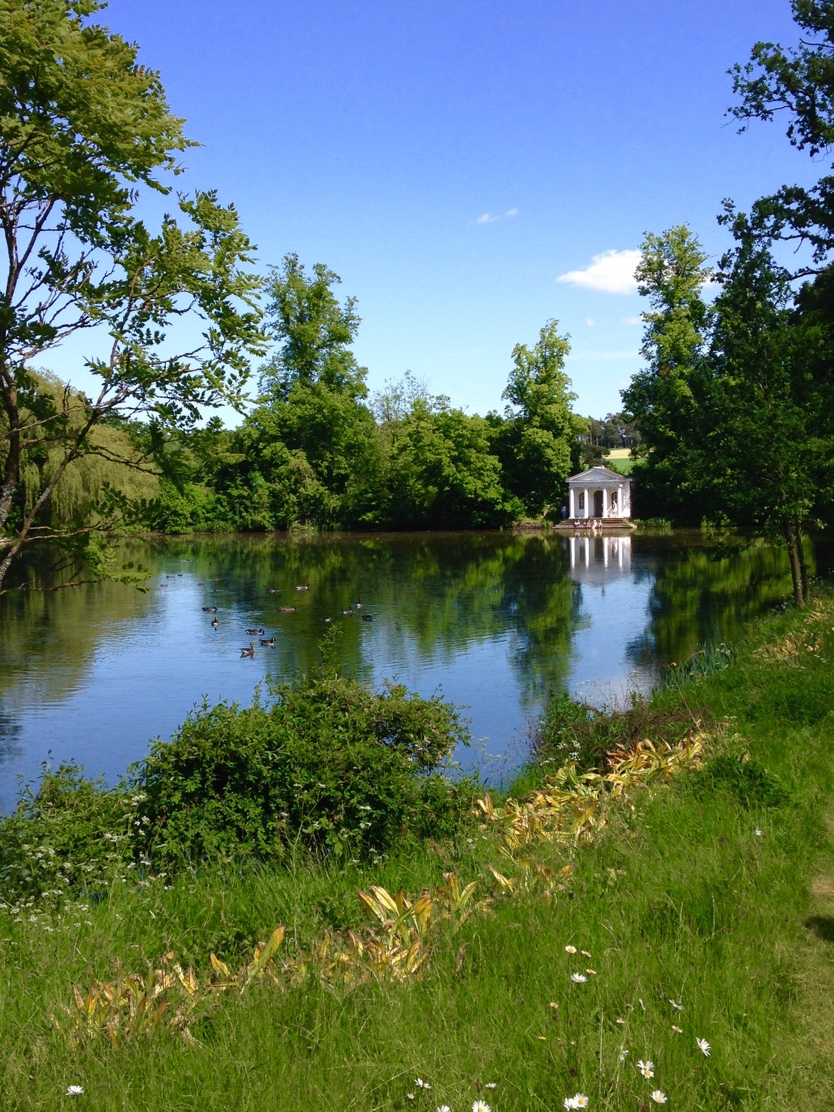


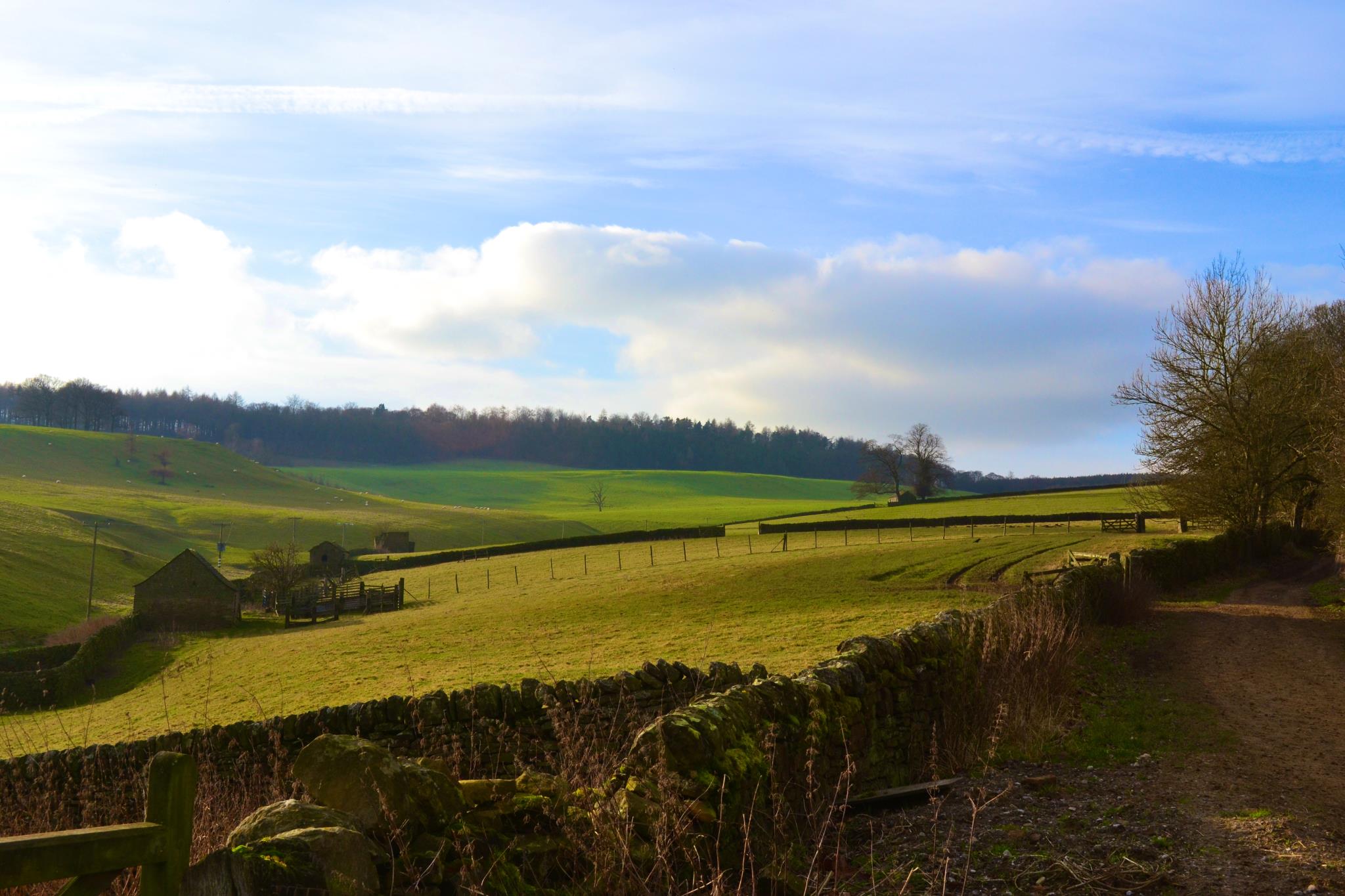


**Set 6 of Soothing Images**


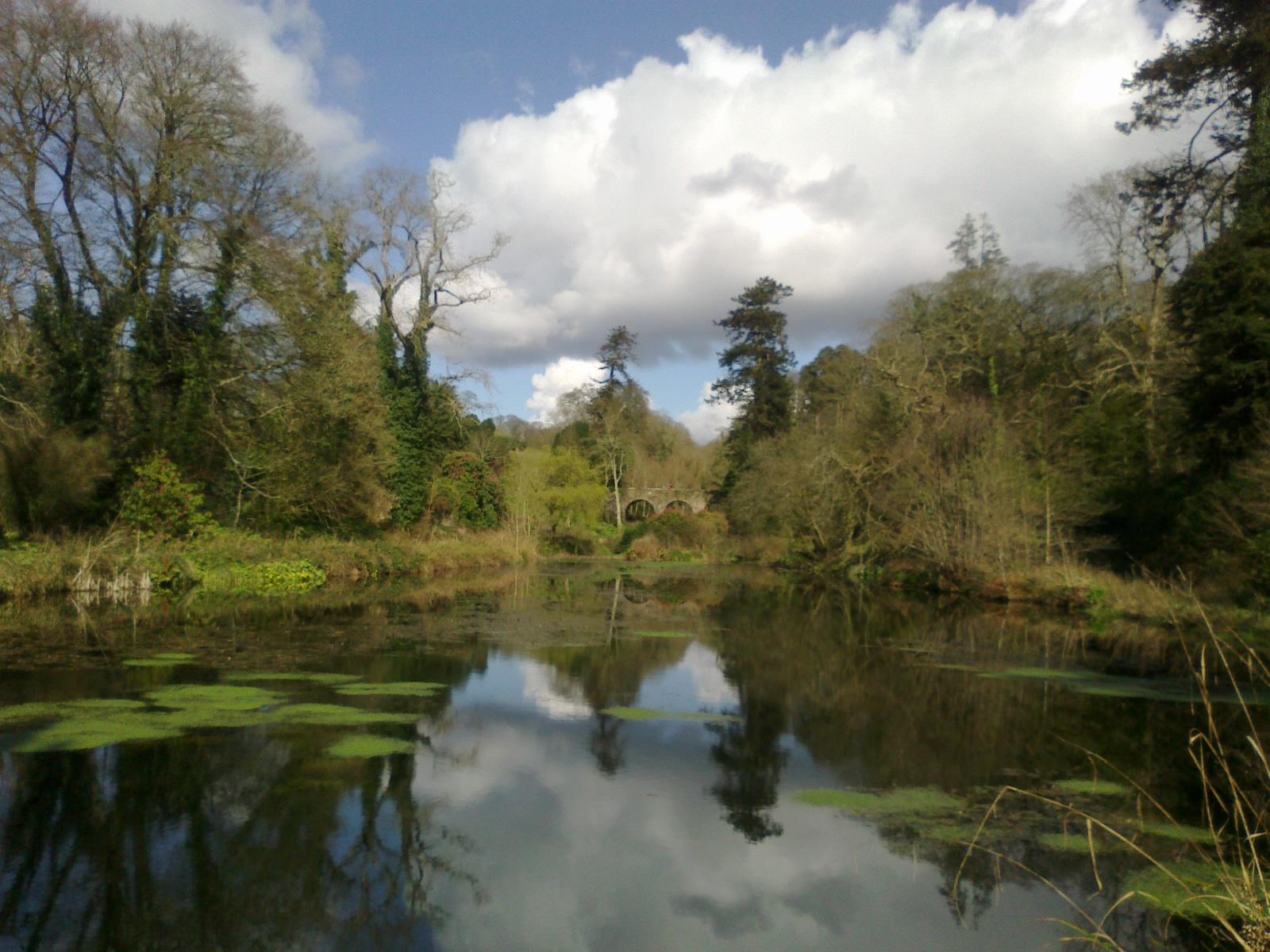


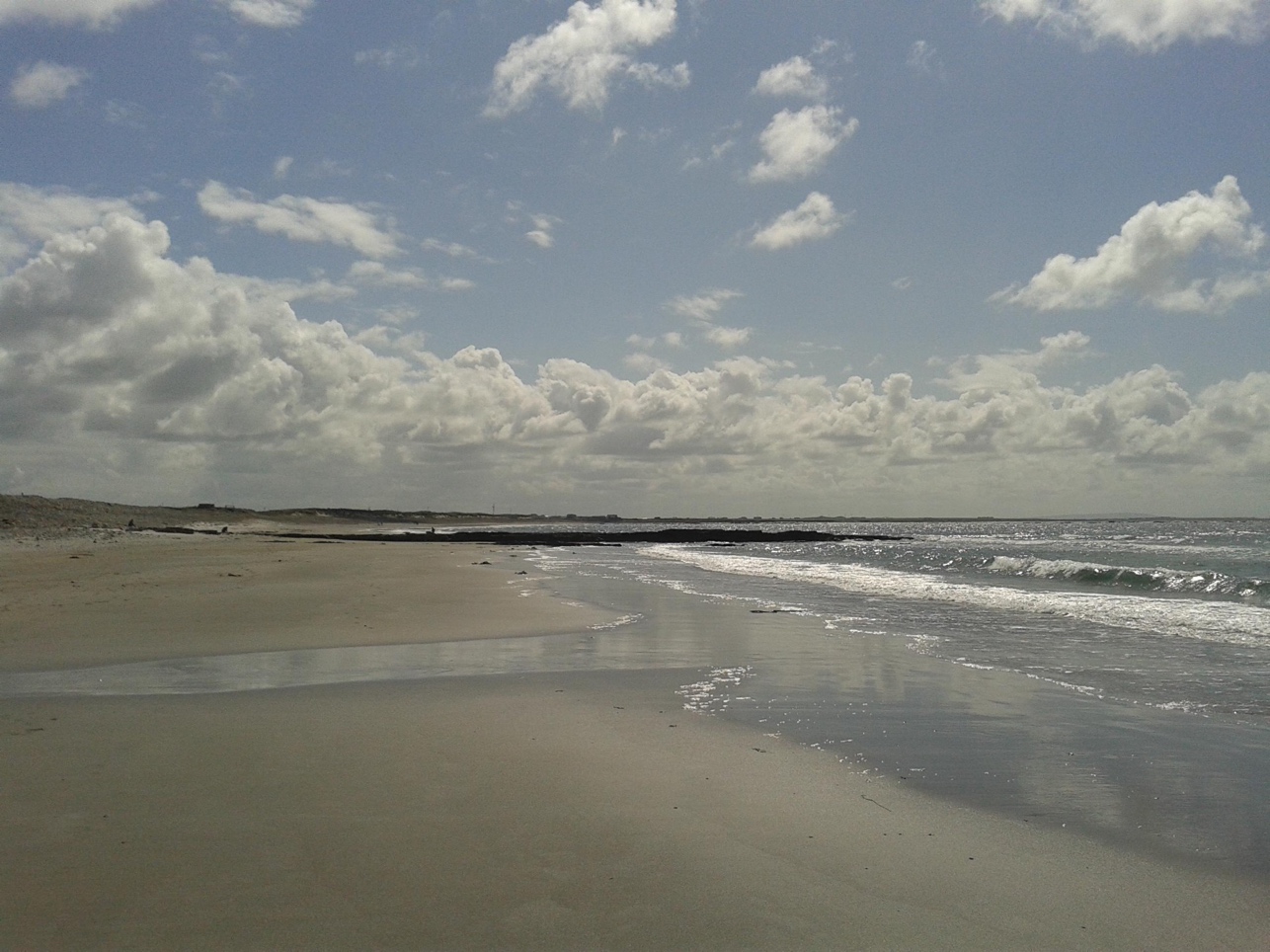


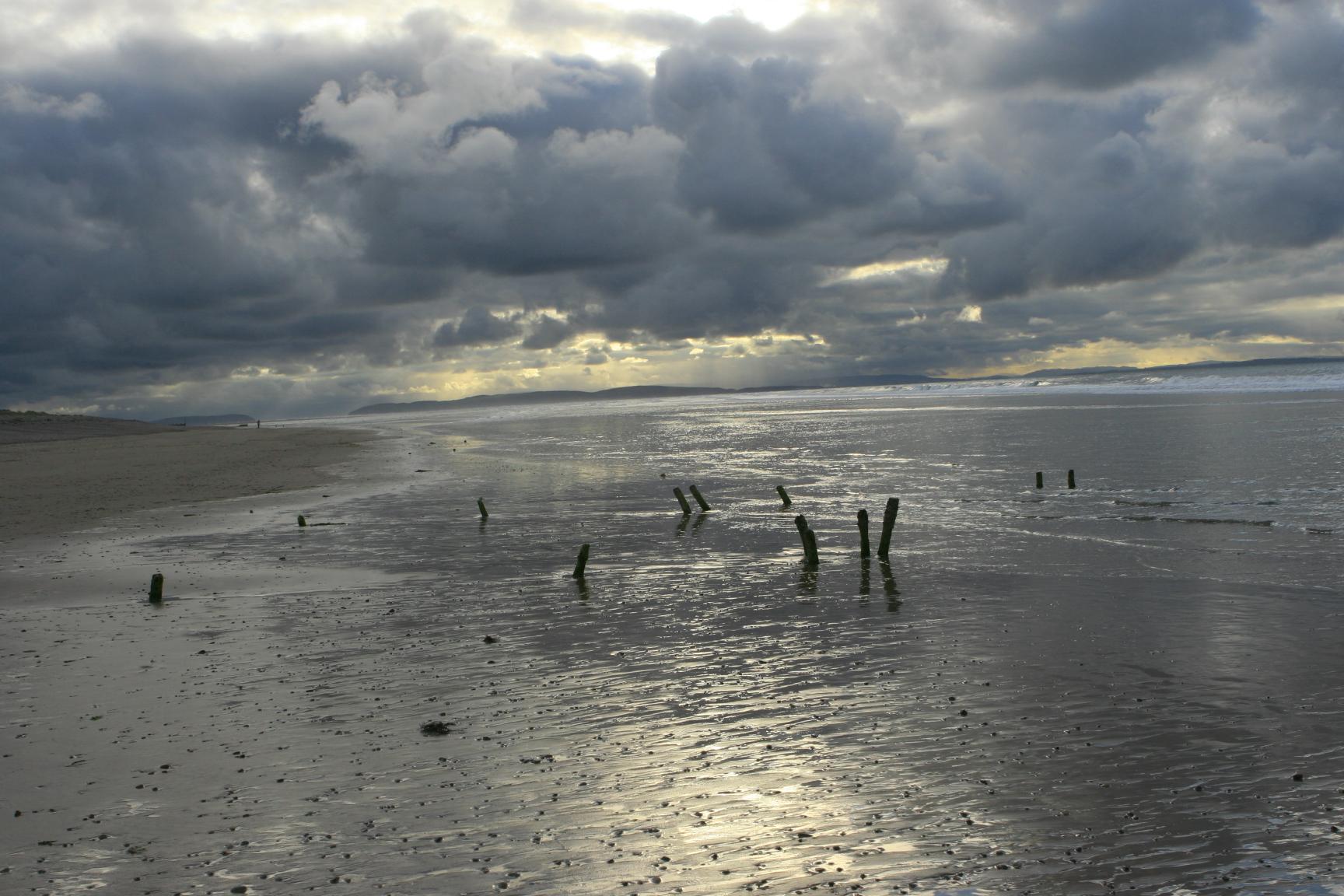


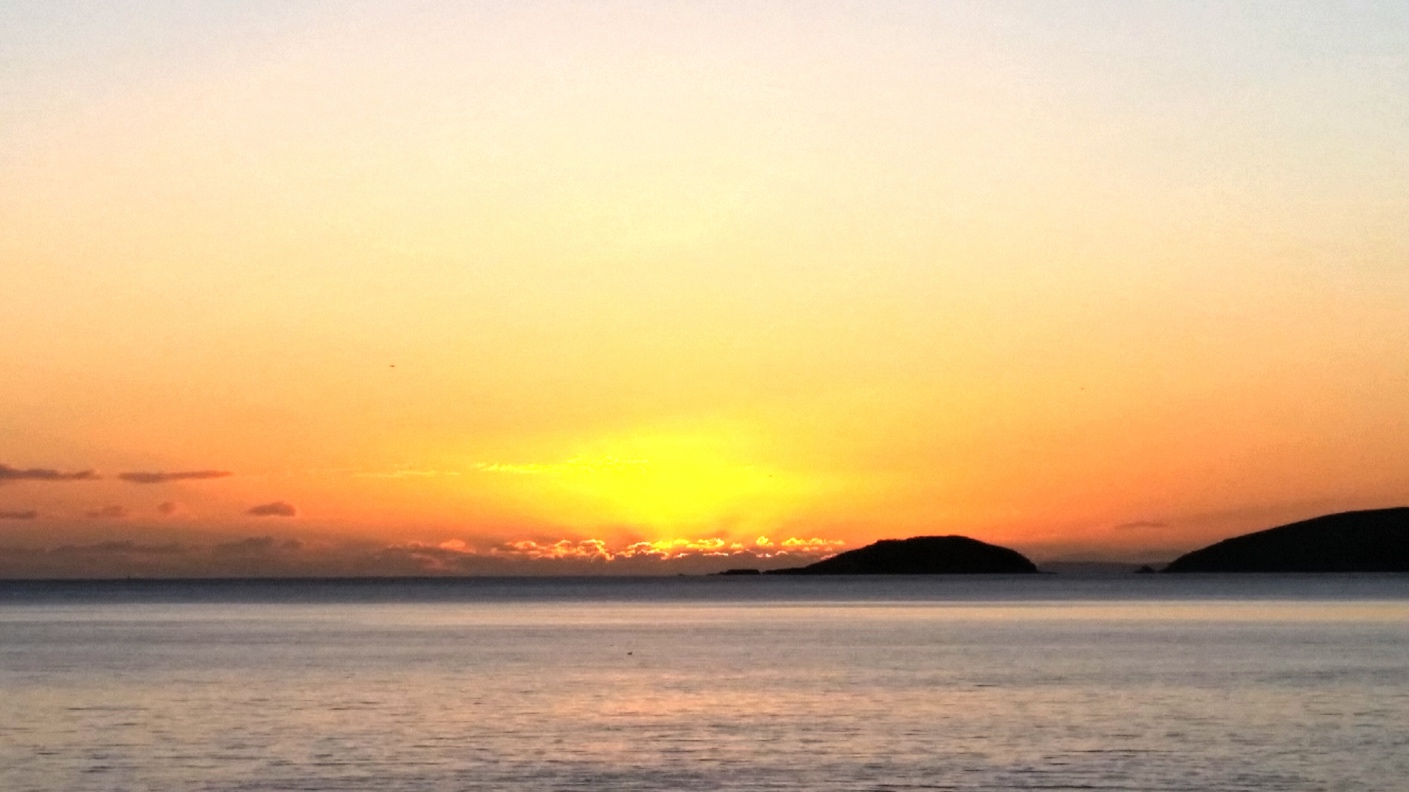


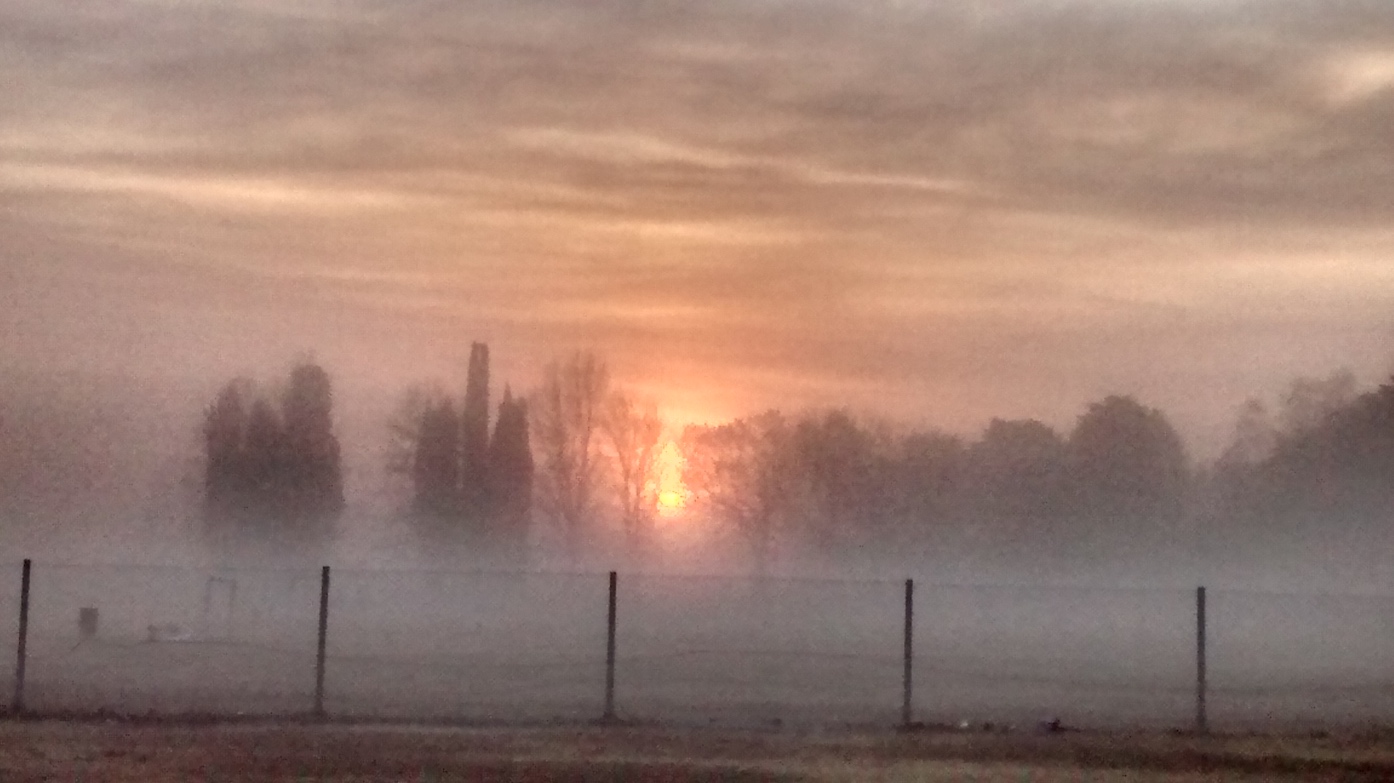


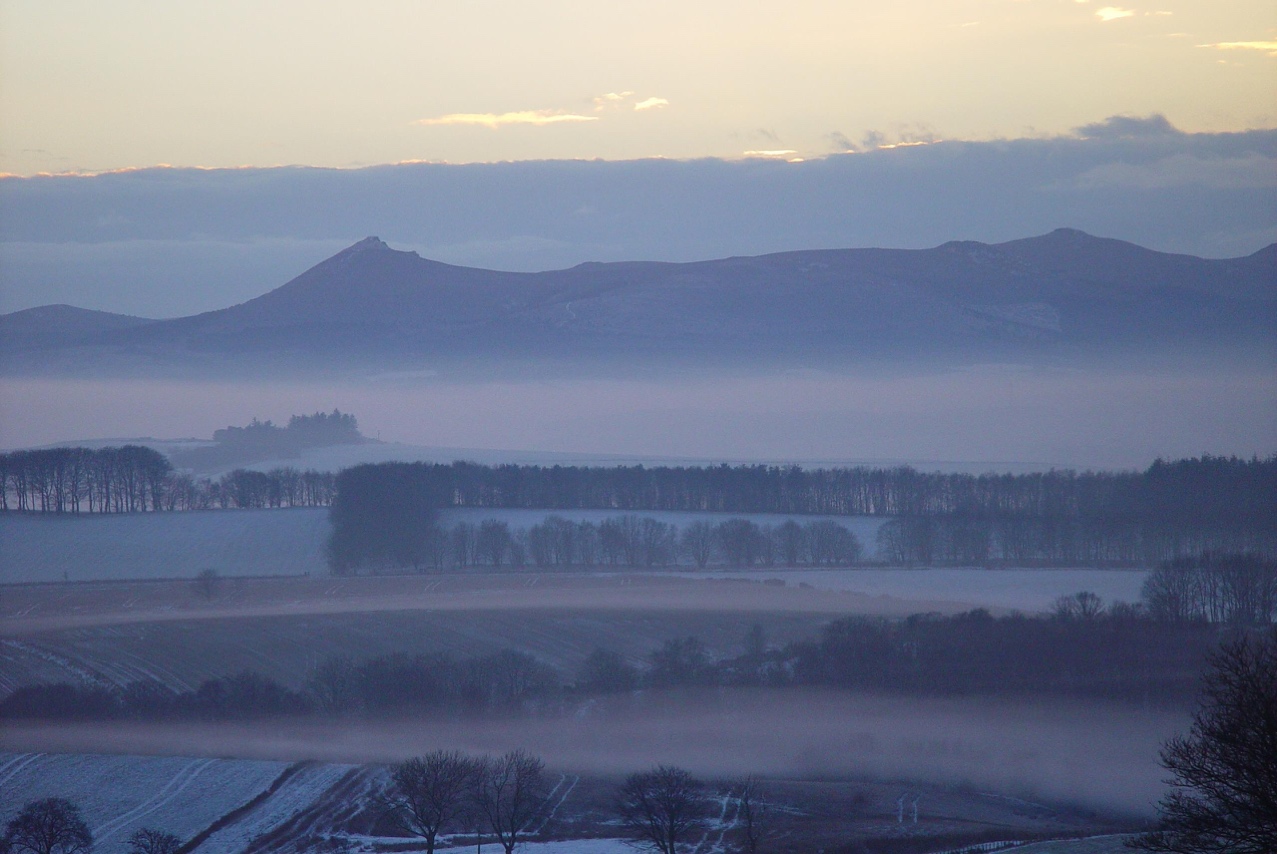


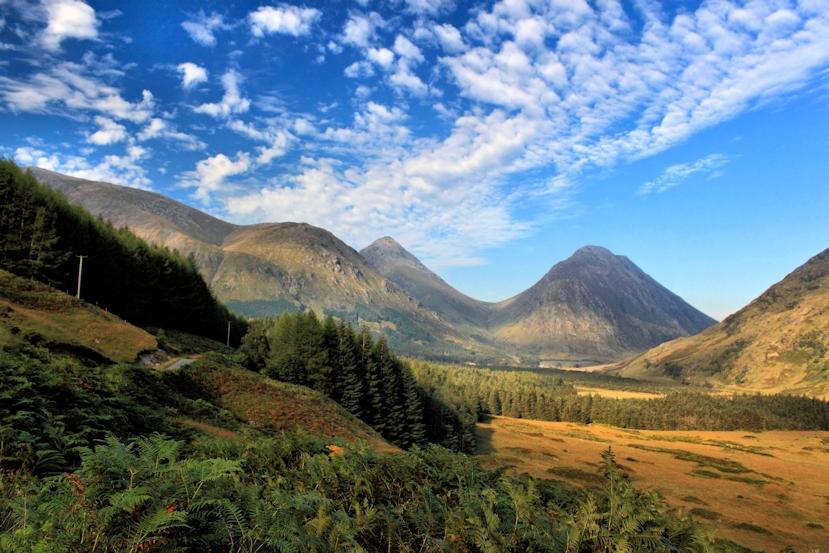


**Additional References**

Bratt, A., Gralberg, I. M., Svensson, I., & Rusner, M. (2020). Gaining the courage to see and accept oneself: Group-based compassion-focussed therapy as experienced by adolescent girls. *Clinical Child Psychology and Psychiatry*, *25*(4), 909–921. https://doi.org/10.1177/1359104520931583

Campbell, F., Blank, L., Cantrell, A. *et al.* (2022). Factors that influence mental health of university and college students in the UK: a systematic review. *BMC Public Health, 22,* 1778. <https://doi.org/10.1186/s12889-022-13943-x>

Chang, E. T., Lai, H. L., Chen, P. W., Hsieh, Y. M., & Lee, L. H. (2012). The effects of music on the sleep quality of adults with chronic insomnia using evidence from polysomnographic and self-reported analysis: A randomized control trial.

*International Journal of Nursing Studies*, *49*(8), 921– 930. https://doi.org/10.1016/j.ijnurstu.2012.02.019

Di Simplicio, M., Appiah-Kusi, E., Wilkinson, P., Watson, P., Meiser-Stedman, C., Kavanagh, D. J., & Holmes, E. A. (2020). Imaginator: A Proof-of-Concept Feasibility Trial of a Brief Imagery-Based Psychological Intervention for Young People Who Self-Harm. *Suicide & life-threatening behavior*, *50*(3), 724–740. https://doi.org/10.1111/sltb.12620

Eisenberg, D., Golberstein, E., & Gollust, S. E. (2007). Help-seeking and access to mental health care in a university student population. *Medical Care*, *45*(7), 594–601. https://doi.org/10.1097/MLR.0b013e31803bb4c1

Eisenberg, D., Downs, M. F., Golberstein, E., & Zivin, K. (2009). Stigma and help seeking for mental health among college students. *Medical care research and review : MCRR*, *66*(5), 522–541. https://doi.org/10.1177/1077558709335173

Eisenberg, D., Speer, N., & Hunt, J. B. (2012). Attitudes and beliefs about treatment among college students with untreated mental health problems. *Psychiatric Services (Washington, D.C.)*, *63*(7), 711–713. https://doi.org/10.1176/appi.ps.201100250

Fodor, L. A., Coteț, C. D., Cuijpers, P., Szamoskozi, Ș., David, D., & Cristea, I. A. (2018). The effectiveness of virtual reality-based interventions for symptoms of anxiety and depression: A meta-analysis. *Scientific Reports, 8*(1), 1-13. https://doi.org/10.1038/s41598-018-28113-6

Gao, L., Zhai, S., Xie, H., Liu, Q., Niu, G., & Zhou, Z. (2022). Big five personality traits and problematic mobile phone use: A meta-analytic review. *Current Psychology: A Journal for Diverse Perspectives on Diverse Psychological Issues, 41*(5), 3093–3110. https://doi.org/10.1007/s12144-020-00817-x

GBD 2019 Mental Disorders Collaborators. (2022). Global, regional, and national burden of

12 mental disorders in 204 countries and territories, 1990-2019: a systematic analysis for the Global Burden of Disease Study 2019. *The Lancet. Psychiatry*, *9*(2), 137–150. https://doi.org/10.1016/S2215-0366(21)00395-3

Holmes, E. A., & Mathews, A. (2010). Mental imagery in emotion and emotional disorders. *Clinical Psychology Review*, *30*(3), 349–362. https://doi.org/10.1016/j.cpr.2010.01.001

Henderson, C., Evans-Lacko, S., & Thornicroft, G. (2013). Mental illness stigma, help seeking, and public health programs. *American Journal of Public Health, 103*(5), 777–780. https://doi.org/10.2105/AJPH.2012.301056

Hughes, G. & Spanner, L. (2019). The University Mental Health Charter. Leeds: Student Minds. Retrieved from: https://www.studentminds.org.uk/uploads/3/7/8/4/3784584/191208_umhc_artwork.pdf

Insight Network Survey. (2020). University Student Mental Health Survey. Retrieved from https://assets.website- files.com/602d05d13b303dec233e5ce3/60305923a557c3641f1a7808_Mental%20Hea lth%20Report%202019%20(2020).pdf

Jo, H., Song, C., & Miyazaki, Y. (2019). Physiological benefits of viewing nature: A systematic review of indoor experiments. *International Journal of Environmental Research and Public Health*, *16*(23), 4739. https://doi.org/10.3390/ijerph16234739

Jo, H., Ikei, H., & Miyazaki, Y. (2022). Physiological and Psychological Benefits of Viewing an Autumn Foliage Mountain Landscape Image among Young Women. *Forests, 13*. 1492. https://doi.org/10.3390/ f13091492

Karsten, J., Penninx, B. W., Riese, H., Ormel, J., Nolen, W. A., & Hartman, C. A. (2012). The state effect of depressive and anxiety disorders on big five personality traits. *Journal of Psychiatric Research*, *46*(5), 644–650. https://doi.org/10.1016/j.jpsychires.2012.01.024

Kotov, R., Gamez, W., Schmidt, F., & Watson, D. (2010). Linking "big" personality traits to anxiety, depressive, and substance use disorders: a meta-analysis. *Psychological Bulletin*, *136*(5), 768–821. https://doi.org/10.1037/a0020327

Legrand, A. C., & Price, M. (2020). Emotionally valenced stimuli impact response inhibition in those with substance use disorder and co-occurring anxiety and depression symptoms. *Journal of Affective Disorders*, *266*, 639–645. https://doi.org/10.1016/j.jad.2020.02.008

Lewis, C., Pearce, J., & Bisson, J. I. (2012). Efficacy, cost-effectiveness and acceptability of

self-help interventions for anxiety disorders: systematic review. *British Journal of*

*Psychiatry*, *200*(1), 15–21. https://doi.org/10.1192/bjp.bp.110.084756

Lewis, E. G., & Cardwell, J. M. (2020). The big five personality traits, perfectionism and their association with mental health among UK students on professional degree programmes. *BMC Psychology*, *8*(1), 54. https://doi.org/10.1186/s40359-020-00423-3

Liu, J., Yan, F., Ma, X., Guo, H. L., Tang, Y. L., Rakofsky, J. J., Wu, X. M., Li, X. Q., Zhu, H., Guo, X. B., Yang, Y., Li, P., Cao, X. D., Li, H. Y., Li, Z. B., Wang, P., & Xu, Q. Y. (2016). Perceptions of public attitudes towards persons with mental illness in Beijing, China: results from a representative survey. *Social Psychiatry and Psychiatric Epidemiology*, *51*(3), 443–453. https://doi.org/10.1007/s00127-015-1125-z

Malouff, J. M., Thorsteinsson, E. B., & Schutte, N. S. (2005). The Relationship Between the Five-Factor Model of Personality and Symptoms of Clinical Disorders: A Meta-Analysis. *Journal of Psychopathology and Behavioral Assessment, 27*(2), 101–114. https://doi.org/10.1007/s10862-005-5384-y

Mokdad, A. H., Forouzanfar, M. H., Daoud, F., Mokdad, A. A., El Bcheraoui, C., Moradi- Lakeh, M., … Murray, C. J. (2016). Global burden of diseases, injuries, and risk factors for young people's health during 1990-2013: a systematic analysis for the Global Burden of Disease Study 2013. *Lancet, 387*(10036), 2383–2401. https://doi.org/10.1016/S0140-6736(16)00648-6

Neff, K. D. (2003b). Self-compassion: An alternative conceptualization of a healthy attitude

toward oneself. *Self and Identity, 2*(2), 85-102. https://doi.org/10.1080/15298860309032

Pile, V., Williamson, G., Saunders, A., Holmes, E. A., & Lau, J. Y. F. (2021). Harnessing emotional mental imagery to reduce anxiety and depression in young people: an integrative review of progress and promise. *The Lancet Psychiatry*, *8*(9), 836–852. https://doi.org/10.1016/S2215-0366(21)00195-4

Pile, V., Smith, P., Leamy, M., Oliver, A., Blackwell, S. E., Meiser-Stedman, R., Dunn, B. D., Holmes, E. A., & Lau, J. Y. F. (2020). Harnessing mental imagery and enhancing memory specificity: Developing a brief early intervention for depressive symptoms in adolescence. *Cognitive Therapy and Research, 45,* 885-901. https://doi.org/10.1007/s10608-020-1 0130-3

Richardson, T., Elliott, P., & Roberts, R. (2017). Relationship between loneliness and mental health in students. *Journal of Public Mental Health, 16*(2), 48-54.

[https://doi.org/10.1108/JPMH-03-2016-0013](https://psycnet.apa.org/doi/10.1108/JPMH-03-2016-0013)

Richardson T, Elliott P, Roberts R, & Jansen M. (2017). A Longitudinal study of financial difficulties and mental health in a national sample of British undergraduate students. *Community Ment Health J, 53*(3), 344–52.

Shin, M. Y., & Shin, W. S. (2019). The effects of viewing natural scenery indoors on middle school students' changes in mood states and concentration. *Journal of People Plants Environment*, *22*(2), 199– 207.

Sweller, J. (1988). Cognitive load during problem solving: Effects on learning. *Cognitive Science, 12*(2), 257-285. https://doi.org/10.1016/0364-0213(88)90023-7

Thomson, C. J., Reece, J. E., & Di Benedetto, M. (2014). The relationship between music- related mood regulation and psychopathology in young people. *Musicae Scientiae, 18*(2), 150-165. <https://doi.org/10.1177/1029864914521422>

Vinograd, M., Williams, A., Sun, M., Bobova, L., Wolitzky-Taylor, K. B., Vrshek-Schallhorn, S., Mineka, S., Zinbarg, R. E., & Craske, M. G. (2020). Neuroticism and interpretive bias as risk factors for anxiety and depression. *Clinical Psychological Science : a Journal of the Association for Psychological Science*, *8*(4), 641–656. https://doi.org/10.1177/2167702620906145

Vidourek, R. A., King, K. A., Nabors, L. A., & Merianos, A. L. (2014). Students' benefits and barriers to mental health help-seeking. *Health Psychology and Behavioral Medicine*, *2*(1), 1009–1022. https://doi.org/10.1080/21642850.2014.963586

Wei, Y., McGrath, P.J., Hayden, J. *et al.* (2015). Mental health literacy measures evaluating knowledge, attitudes, and help-seeking: a scoping review. *BMC Psychiatry,* *15,* 291. https://doi.org/10.1186/s12888-015-0681-9

Yang, W., Lin, L., Zhu, W., & Liang S. (2015). An introduction to mental health services at universities in China. Mental Health & Prevention. *Mental Health and Prevention, 3*(1-2), 11-16. https://doi.org/10.1016/j.mhp.2015.04.001

Yu, T., Xu, J., Jiang, Y. *et al.* (2022). School educational models and child mental health among K-12 students: a scoping review. *Child Adolesc Psychiatry Ment Health,* *16,* 32. <https://doi.org/10.1186/s13034-022-00469-8>

Yanguas, J., Pinazo-Henandis, S., & Tarazona-Santabalbina, F. J. (2018). The complexity of loneliness. *Acta bio-medica : Atenei Parmensis*, *89*(2), 302–314. <https://doi.org/10.23750/abm.v89i2.7404>

Yulia Korzhina, Jessica Hemberg, Pia Nyman-Kurkiala & Lisbeth Fagerström (2022). Causes of involuntary loneliness among adolescents and young adults: an integrative review, *International Journal of Adolescence and Youth, 27*,1, 493-514, doi: 10.1080/02673843.2022.2150088

Nakshine, V. S., Thute, P., Khatib, M. N., & Sarkar, B. (2022). Increased screen time as a cause of declining physical, psychological health, and sleep patterns: a literary review. *Cureus*, *14*(10), e30051. <https://doi.org/10.7759/cureus.30051>

Smith, R., Wuthrich, V., Johnco, C., & Belcher, J. (2021). Effect of group cognitive behavioural therapy on loneliness in a community sample of older adults: a secondary analysis of a randomized controlled trial. *Clinical Gerontologist*, *44*(4), 439–449. <https://doi.org/10.1080/07317115.2020.1836105>

Colizzi, M., Lasalvia, A. & Ruggeri, M. (2020). Prevention and early intervention in youth mental health: is it time for a multidisciplinary and trans-diagnostic model for care?. *Int J Ment Health Syst*, *14*(23). <https://doi.org/10.1186/s13033-020-00356-9>

Masi, C. M., Chen, H. Y., Hawkley, L. C., & Cacioppo, J. T. (2011). A meta-analysis of interventions to reduce loneliness. *Personality and social psychology review : an official journal of the Society for Personality and Social Psychology, Inc*, *15*(3), 219–266. <https://doi.org/10.1177/1088868310377394>

Gunnes, M., Løe, I. C., & Kalseth, J. (2024). Exploring the impact of information and communication technologies on loneliness and social isolation in community-dwelling older adults: a scoping review of reviews. *BMC geriatrics*, *24*(1), 215. <https://doi.org/10.1186/s12877-024-04837-1>

Hu, J., Zhang, Z., Jiang, K., and Chen, W. (2019). Getting ahead, getting along, and getting prosocial: examining extraversion facets, peer reactions, and leadership emergence. *J. Appl. Psychol.* 11, 1369–1386. doi: 10.1037/apl0000413

Deng, Y., Chen, H., & Yao, X. (2021). Curvilinear effects of extraversion on socialization outcomes among chinese college students. *Frontiers in Psychology*, *12*, 652834. <https://doi.org/10.3389/fpsyg.2021.652834>

Zee, M., Koomen, H., and Van der Veen, I. (2013). Student-teacher relationship quality and academic adjustment in upper elementary school: the role of student personality. *J. Sch. Psychol.* 51, 517–533. doi: 10.1016/j.jsp.2013.05.003

Bucher, M. A., Suzuki, T., & Samuel, D. B. (2019). A meta-analytic review of personality traits and their associations with mental health treatment outcomes. *Clinical Psychology Review*, *70*, 51–63. <https://doi.org/10.1016/j.cpr.2019.04.002>

Roberts, B. W., Luo, J., Briley, D. A., Chow, P. I., Su, R., & Hill, P. L. (2017). A systematic review of personality trait change through intervention. *Psychological Bulletin*, *143*(2), 117–141. <https://doi.org/10.1037/bul0000088>

Searight, H. R., & Montone, K. (2017). Profile of Mood States. *Encyclopaedia of Personality and Individual Differences*, 1–6. <https://doi.org/10.1007/978-3-319-28099-8_63-1>

Watson, D., & Walker, L. M. (1996). The long-term stability and predictive validity of trait measures of affect. *Journal of Personality and Social Psychology*, *70*(3), 567–577. <https://doi.org/10.1037//0022-3514.70.3.567>

Plys, E., & Desrichard, O. (2020). Associations Between Positive and Negative Affect and the Way People Perceive Their Health Goals. *Frontiers in Psychology*, *11*, 334. <https://doi.org/10.3389/fpsyg.2020.00334>

Valentina Rossi Valentina.Rossi@UGent.be & Gilles Pourtois (2012) Transient state-dependent fluctuations in anxiety measured using STAI, POMS, PANAS or VAS: a comparative review. *Anxiety, Stress, & Coping, 25*(6), 603-645, doi: 10.1080/10615806.2011.582948

Boyle, G. J., Helmes, E., Matthews, G., & Izard, C. E. (2015, January 1). *Chapter 8 - Measures of Affect Dimensions* (G. J. Boyle, D. H. Saklofske, & G. Matthews, Eds.). ScienceDirect; Academic Press. https://www.sciencedirect.com/science/article/pii/B9780123869159000085

Chaudhry, S., Tandon, A., Shinde, S., & Bhattacharya, A. (2024). Student psychological well-being in higher education: The role of internal team environment, institutional, friends and family support and academic engagement. *PloS one*, *19*(1), e0297508. <https://doi.org/10.1371/journal.pone.0297508>

Mofatteh M. (2020). Risk factors associated with stress, anxiety, and depression among university undergraduate students. *AIMS Public Health*, *8*(1), 36–65. <https://doi.org/10.3934/publichealth.2021004>

Campbell, F., Blank, L., Cantrell, A., Baxter, S., Blackmore, C., Dixon, J., & Goyder, E. (2022). Factors that influence mental health of university and college students in the UK: a systematic review. *BMC Public Health*, *22*(1), 1778. <https://doi.org/10.1186/s12889-022-13943-x>

Letzring, T. D., & Adamcik, L. A. (2015). Personality traits and affective states: Relationships with and without affect induction. Personality and Individual Differences, 75, 114–120. [https://doi.org/10.1016/j.paid.2014.11.011](https://psycnet.apa.org/doi/10.1016/j.paid.2014.11.011)

Lahey B. B. (2009). Public health significance of neuroticism. *The American Psychologist*, *64*(4), 241–256. <https://doi.org/10.1037/a0015309>

Smillie, L. D., DeYoung, C. G., & Hall, P. J. (2015). Clarifying the relation between extraversion and positive affect. *Journal of Personality*, *83*(5), 564–574. <https://doi.org/10.1111/jopy.12138>

Davidson, E. J., Taylor, C. T., Ayers, C. R., Quach, N. E., Tu, X. M., & Lee, E. E. (2022). The Relationship between loneliness and positive affect in older adults. *The American Journal of Geriatric Psychiatry: official journal of the American Association for Geriatric Psychiatry*, *30*(6), 678–685. <https://doi.org/10.1016/j.jagp.2021.11.002>

Yanguas, J., Pinazo-Henandis, S., & Tarazona-Santabalbina, F. J. (2018). The complexity of loneliness. *Acta bio-medica : Atenei Parmensis*, *89*(2), 302–314. <https://doi.org/10.23750/abm.v89i2.7404>

Shi, W., Shen, Z., Wang, S., & Hall, B. J. (2020). Barriers to professional mental health help-seeking among Chinese adults: A Systematic Review. *Frontiers in Psychiatry*, *11*, 442. <https://doi.org/10.3389/fpsyt.2020.00442>

Mushtaq, R., Shoib, S., Shah, T., & Mushtaq, S. (2014). Relationship between loneliness, psychiatric disorders and physical health ? A review on the psychological aspects of loneliness. *Journal of clinical and diagnostic research : JCDR*, *8*(9), WE01–WE4. <https://doi.org/10.7860/JCDR/2014/10077.4828>
