# Supplementary material for: Effects of the Soothe Vision well-being tool on university students’ mood: a pilot study
Source: Curr Psychol. 2025 Mar 31;44(10):9112–28. doi: 10.1007/s12144-025-07649-7 (PMC12144047; doi:10.1007/s12144-025-07649-7)
Supplement: Supplementary file 2 — Supplementary file2 (DOCX 16 KB) [file 12144_2025_7649_MOESM2_ESM.docx]

**Appendix-II:**

**RESULTS -- ANCOVA**

Due to significant differences in baseline scores of extraversion and loneliness among the groups, separate Analyses of Covariance (ANCOVAs) were performed. The findings suggested that loneliness or extraversion scores did not have a significant influence on mood changes. The results of loneliness and extraversion on mood changes are provided below:

**Loneliness and Positive Affect:**

The results of the ANCOVA revealed that loneliness scores did not significantly impact the main effect of time, F (1, 149) = 2.15, p = 0.14, d = 0.014, or group, F (1, 149) = 0.92, p = 0.76, d = 0.001. Moreover, loneliness as a covariate did not affect the Time × Group interaction, F (1, 149) = 0.37, p = 0.55, d = 0.002.

**Loneliness and Negative Affect:**

The results of the ANCOVA showed that loneliness scores did not significantly impact the main effect of time, F (1, 149) = 2.05, p = 0.15, d = 0.014, or group, F (1, 149) = 0.06, p = 0.94, d = 0.001. Moreover, loneliness as a covariate did not affect the Time × Group interaction, F (1, 149) = 0.11, p = 0.75, d = 0.001.

**Loneliness and Depressive Mood states:**

The loneliness scores did not significantly impact the main effect of time, F (1, 149) = 0.005, p = 0.95, d = 0.000, or group, F (1, 149) = 0.002, p = .95, d = .000. Moreover, loneliness as a covariate did not affect the Time × Group interaction, F (1, 149) = 0.14, p = 0.55, d = 0.001.

**Loneliness and Anxious Mood states:**

The results of the ANCOVA revealed that loneliness scores did not significantly impact the main effect of time, F (1, 149) = 5.87, p = 0.02, d = 0.038, or group, F (1, 149) = 0.140, p = 0.71, d = .001. Moreover, loneliness as a covariate did not affect the Time × Group interaction, F (1, 149) = 0.36, p = 0.71, d = 0.002.

**Loneliness and Serenity Affect:**

The results showed that loneliness scores did not significantly impact the main effect of time, F (1, 149) = 0.006, p = 0.94, d = 0.000, or group, F (1, 149) = 1.41, p = 0.24, d = .009. Moreover, loneliness as a covariate did not affect the Time × Group interaction, F (1, 149) = 0.010, p = 0.92, d = 0.000.

**Extraversion and Positive Affect:**

The results showed that extraversion scores did not significantly impact the main effect of time, F (1, 149) = 3.49, p = 0.06, d = 0.023, or group, F (1, 149) = 0.55 p = 0.462, d = 0.004. Moreover, loneliness as a covariate did not affect the Time × Group interaction, F (1, 149) = 0.49, p = 0.49, d = 0.003.

**Extraversion and Negative Affect:**

The results of the ANCOVA revealed that extraversion scores did not significantly impact the main effect of time, F (1, 149) = 3.49, p = 0.06, d = 0.023, or group, F (1, 149) = 0.402, p = 0. 53, d = 0. 003. Moreover, loneliness as a covariate did not affect the Time × Group interaction, F (1, 149) = 0.121, p = 0.72, d = 0.001.

**Extraversion and Depressive Mood states:**

The results of the ANCOVA revealed that extraversion scores did not significantly impact the main effect of time, F (1, 149) = 9.44, p = 0.31, d = 0.06, or group, F (1, 149) = 0. 97, p = . 33, d = .000. Moreover, loneliness as a covariate did not affect the Time × Group interaction, F (1, 149) = 0.022 p = 0.88, d = 0.001.

**Extraversion and Anxious Mood states:**

The results of the ANCOVA revealed that extraversion scores did not significantly impact the main effect of time, F (1, 149) = 7.84, p = 0.61, d = 0.050, or group, F (1, 149) = 0. 931, p = 0. 34, d = 0. 006. Moreover, loneliness as a covariate did not affect the Time × Group interaction, F (1, 149) = 0.75, p = 0.389, d = 0.005.

**Extraversion and Serenity Affect:**

The results of the ANCOVA revealed that extraversion scores did not significantly impact the main effect of time, F (1, 149) = 0.006, p = 0.94, d = 0.000, or group, F (1, 149) = 0.000, p = 0.99, d = 0.000. Moreover, loneliness as a covariate did not affect the Time × Group interaction, F (1, 149) = 0.041, p = 0.84, d = 0.000
